# Association between the XPG Asp1104His and XPF Arg415Gln Polymorphisms and Risk of Cancer: A Meta-Analysis

**DOI:** 10.1371/journal.pone.0088490

**Published:** 2014-05-06

**Authors:** Xiao-Feng He, Li-Rong Liu, Wu Wei, Yi Liu, Jiao Su, Su-Lan Wang, Xu-Liang Shen, Xian-Bin Yang

**Affiliations:** 1 Department of Research, Peace Hospital of Changzhi Medical College, Changzhi, China; 2 Department of Clinical Biochemistry, Affiliated Hospital of Guiyang Medical University, Guiyang, China; 3 Department of Hematology, Peace Hospital of Changzhi Medical College, Changzhi, China; 4 Department of Neurosurgery, Nanfang Hospital, Southern Medical University, Guangzhou, China; 5 Department of Biological Chemistry, Changzhi Medical College, Changzhi, China; Institut Jacques Monod, France

## Abstract

**Backgroud:**

The XPG (xeroderma pigmentosum type G) Asp1104His and XPF (xeroderma pigmentosum type F) Arg415Gln polymorphisms had been implicated in cancer susceptibility. The previous published data on the association between XPG Asp1104His and XPF Arg415Gln polymorphisms and cancer risk remained controversial.

**Methodology/Principal Findings:**

To derive a more precise estimation of the association between the XPG Asp1104His and XPF Arg415Gln polymorphisms and overall cancer risk, we performed a meta-analysis to investigate the association between cancer susceptibility and XPG Asp1104His (32,162 cases and 39,858 controls from 66 studies) and XPF Arg415Gln polymorphisms (17,864 cases and 20,578 controls from 32 studies) in different inheritance models. We used odds ratios with 95% confidence intervals to assess the strength of the association. Overall, significantly elevated cancer risk was found when all studies were pooled into the meta-analysis of XPG Asp1104His (dominant model: OR = 1.05, 95% CI = 1.00–1.10; Asp/His vs. Asp/Asp: OR = 1.06, 95% CI = 1.01–1.11). In the further stratified and sensitivity analyses, significantly decreased lung cancer risk was found for XPF Arg415Gln (dominant model: OR = 0.82, 95% CI = 0.71–0.96; Arg/Gln versus Arg/Arg: OR = 0.83, 95% CI = 0.71–0.97; additive model: OR = 0.83, 95% CI = 0.72–0.95) and significantly increased other cancer risk was found among hospital-based studies for XPG Asp1104His (dominant model: OR = 1.23, 95% CI = 1.02–1.49).

**Conclusions/Significance:**

In summary, this meta-analysis suggests that XPF Arg415Gln polymorphism may be associated with decreased lung cancer risk and XPG Asp1104His may be a low-penetrant risk factor in some cancers development. And larger scale primary studies are required to further evaluate the interaction of XPG Asp1104His and XPF Arg415Gln polymorphisms and cancer risk in specific populations.

## Introduction

DNA repair systems play critical roles in protecting cells against mutations and are essential for maintaining the genome integrity. Certain common genetic polymorphisms within the genes involved in DNA damage responses may contribute to the development of cancer and be associated with an increased risk of the disease. Because reduced DNA repair capacity may cause genetic instability and carcinogenesis, genes involved in DNA repair have been proposed as candidate cancer susceptibility genes [1]. Nucleotide excision repair (NER) is a crucial DNA repair mechanism, which counteracts the consequences of mutagenic exposure of cells [2].

The NER pathway consists of >30 proteins involved in DNA damage recognition, incision, DNA ligation and resynthesis. Seven XP(xeroderma pigmentosum) complementation groups have been identified, from XPA to XPG, representing the malfunctioning proteins in the NER mechanism [3]. The XPG (xeroderma pigmentosum type G), one important component of the NER pathway, encodes a structure-specific endonuclease catalyzing 3′ incision and involves the subsequent 5′ incision by ERCC1-XPF heterodimer [4], [5]. It has been observed that there is a relationship between the SNP in exon 15 (G3507C, Asp1104His) and cancer susceptibility. ERCC4/XPF (Arg-to-Gln substitution in codon 415 of exon 8, rs1800067) forms a tight complex with ERCC1 to incise 5′ to the damage site recognized and repaired by NER [6]. The XPF gene encodes a protein which, together with ERCC1, creates the 5′ endonuclease [7].

To date, a number of molecular epidemiological studies have been done to evaluate the association between XPG Asp1104His and XPF Arg415Gln polymorphisms and different types of cancer risk in diverse populations [8]–[83]. However, the results were inconsistent or even contradictory, partially because of the possible small effect of the polymorphism on cancer risk and the relatively small sample size in each of published study. In addition, two recent meta-analyses have studied the association between XPG Asp1104His and XPF Arg415Gln and risk of cancer. However, many published studies were not included in the two recent meta-analyses [84], [85]. Therefore, we performed a comprehensive meta-analysis by including the most recent and relevant articles to identify statistical evidence of the association between XPG Asp1104His and XPF Arg415Gln polymorphisms and risk of all cancers that have been investigated. Meta-analysis is an outstanding tool for summarizing the different studies. It can not only overcome the problem of small size and inadequate statistical power of genetic studies of complex traits, but also can provide more reliable results than a single case–control study.

## Materials and Methods

### Identification and eligibility of relevant studies

A comprehensive literature search was performed using the PubMed and Medline database for relevant articles published (the last search update was Sep 5, 2013) with the following key words “XPG”, “ERCC5”, “XPF”, “ERCC4”, “polymorphism”, “Variant” or “Mutation”, and “Cancer” or “Carcinoma.” In addition, studies were identified by a manual search of the reference lists of reviews and retrieved studies. We included all the case–control studies and cohort studies that investigated the association between XPG Asp1104His and XPF Arg415Gln polymorphisms and cancer risk with genotype data. All eligible studies were retrieved, and their bibliographies were checked for other relevant publications. When the same sample was used in several publications, only the most complete study was considered for further analysis.

### Inclusion criteria

The included studies needed to have met the following criteria:: (1) only the case–control studies or cohort studies were considered, (2) evaluated the XPG Asp1104His and XPF Arg415Gln polymorphisms and the risk of cancer, and (3) the genotype distribution of the polymorphisms in cases and controls were described in details and the results were expressed as odds ratio (OR) and corresponding 95% confidence interval (95% CI). Major reasons for exclusion of studies were as follows: (1) not for cancer research, (2) only case population, and (3) duplicate of previous publication.

### Data extraction

Information was carefully extracted from all eligible studies independently by two investigators according to the inclusion criteria listed above. The following data were collected from each study: first author's name, year of publication, country of origin, ethnicity, source of controls, sample size, and numbers of cases and controls in the XPG Asp1104His and XPF Arg415Gln genotypes whenever possible. Ethnicity was categorized as “Caucasian,” “African,” (including African Americans) and “Asian.” Two studies were carried out with Hispanic ethnic groups. When one study did not state which ethnic groups was included or if it was impossible to separate participants according to phenotype, the sample was termed as “mixed population.” Meanwhile, studies investigating more than one kind of cancer were counted as individual data set only in subgroup analyses by cancer type. We did not define any minimum number of patients to include in this meta-analysis. In case of articles reported different ethnic groups and different countries or locations, we considered them different study samples for each category cited above.

### Statistical analysis

Crude odds ratios (ORs) together with their corresponding 95% CIs were used to assess the strength of association between the XPG Asp1104His and XPF Arg415Gln polymorphisms and the risk of cancer. The pooled ORs were performed for co-dominant model (XPG Asp1104His: His/His versus Asp/Asp and Asp/His versus Asp/Asp, XPF Arg415Gln: Gln/Gln versus Arg/Arg and Arg/Gln versus Arg/Arg); dominant model (XPG Asp1104His: Asp/His+His/His versus Asp/Asp, XPF Arg415Gln: Arg/Gln+Gln/Gln versus Arg/Arg); recessive model (XPG Asp1104His: His/His versus Asp/His+Asp/Asp, XPF Arg415Gln: Gln/Gln versus Arg/Gln+Arg/Arg); and additive model (XPG Asp1104His: His versus Asp, XPF Arg415Gln: Gln versus Arg), respectively. Between-study heterogeneity was assessed by calculating *Q*-statistic (Heterogeneity was considered statistically significant if *P*<0.10) [86] and quantified using the *I^2^* value, a value that describes the percentage of variation across studies that are due to heterogeneity rather than chance, where *I^2^* = 0% indicates no observed heterogeneity, with 25% regarded as low, 50% as moderate, and 75% as high [87]. If results were not heterogeneous, the pooled ORs were calculated by the fixed-effect model (we used the *Q*-statistic, which represents the magnitude of heterogeneity between-studies) [88]. Otherwise, a random-effect model was used (when the heterogeneity between-studies were significant) [89]. In addition to the comparison among all subjects, we also performed stratification analyses by cancer type (if one cancer type contained less than three individual studies, it was combined into the “other cancers” group), Moreover, the extent to which the combined risk estimate might be affected by individual studies was assessed by consecutively omitting every study from the meta-analysis (leave-one-out sensitivity analysis). This approach would also capture the effect of the oldest or first positive study (first study effect). In addition, we also ranked studies according to sample size, and then repeated this meta-analysis. Sample size was classified according to a minimum of 200 participants and those with fewer than 200 participants. The cite criteria were previously described [90]. Last, sensitivity analysis was also performed, excluding studies whose allele frequencies in controls exhibited significant deviation from the Hardy–Weinberg equilibrium (HWE), given that the deviation may denote bias. HWE was calculated by using the goodness-of-fit test, and deviation was considered when *P*<0.05. Begg's funnel plots [91] and Egger's linear regression test [92] were used to assess publication bias. If publication bias existed, the Duval and Tweedie nonparametric “trim and fill” method was used to adjust for it [93]. A meta-regression analysis was carried out to identify the major sources of between-studies variation in the results, using the log of the ORs from each study as dependent variables, and cancer type, ethnicity, sample size, HWE, and source of controls as the possible sources of heterogeneity. All of the calculations were performed using STATA version 10.0 (STATA Corporation, College Station, TX).

## Results

### Eligible studies and meta-analysis databases

**Figure 1:**
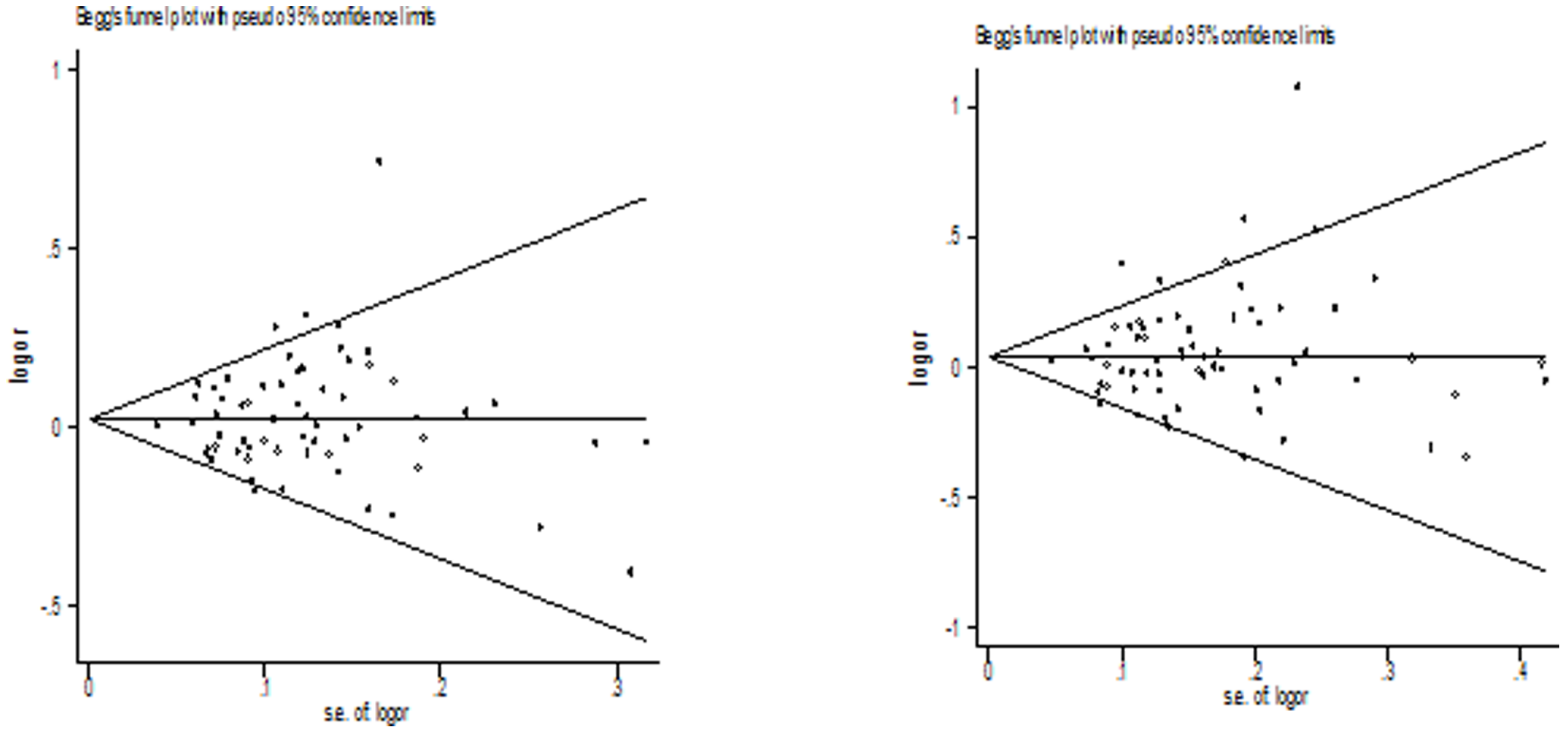
Study flow chart explaining the selection of the 72 eligible articles included in the meta-analysis.

**Fig. 1** graphically illustrates the trial flow chart. A total of 236 articles regarding XPG Asp1104His and XPF Arg415Gln polymorphisms with respect to cancer were identified. After screening the titles and abstracts, 160 articles were excluded because they were review articles, case reports, other polymorphisms of CYP1A1, or irrelevant to the current study. In addition, of these published articles, 4 publications [76]–[79] were excluded because of their populations overlapped with another 3 included studies [40], [44], [68]. Five publications [17], [20], [40], [41], [57] including different case–control groups should be considered as two separate studies each. As summarized in **Table 1**, 72 publications with 98 case–control studies were selected among the meta-analysis, including 32,162 cases and 39,858 controls for XPG Asp1104His (66 studies from 62 publications) and 17,864 cases and 20,578 controls for XPF Arg415Gln (32 studies from 29 publications). Among these studies, for XPG Asp1104His, there were 7 bladder cancer studies, 11 breast cancer studies, 7 colorectal cancer studies, 5 head and neck cancer studies, 7 lung cancer studies, 4 non-Hodgkin lymphoma studies, 3 glioma studies, 8 melanoma studies, and 14 studies with the “other cancers”. There were 10 breast cancer studies, 3 lung cancer studies, 4 head and neck cancer studies, 4 colorectal cancer, 3 glioma studies, and 8 studies with the “other cancers” for XPF Arg415Gln. All of the cases were pathologically confirmed.

**Table 1:** Main characteristics of all studies included in the meta-analysis.

First author/year	Country	Ethnicity	Cancer type	SC	XPG Asp1104His (Case/control)			XPF Arg415Gln (Case/control)			HWE
Smith [8] 2003	USA	Caucasian	Breast	HB	NA	NA	NA	217/236	29/32	7/0	Yes
Kumar [9] 2003	Filand	Caucasian	Breast	HB	108/182	96/107	16/19	NA	NA	NA	Yes
Jeon [10] 2003	Korea	Asian	Lung	HB	58/90	164/132	88/89	NA	NA	NA	No
Sanyal [11] 2004	Swede	Caucasian	Bladder	NA	182/173	109/91	8/20	NA	NA	NA	Yes
Blankenburg [12] 2005	German	Caucasian	Melanoma	HB	184/232	100/124	9/18	NA	NA	NA	Yes
Weiss [13] 2005	USA	Mixed	Endometrial	PB	215/250	134/148	22/22	316/369	54/49	1/2	Yes
Shen [14] 2005	China	Asian	Lung	PB	26/25	52/46	38/38	NA	NA	NA	Yes
Bigler [15] 2005	USA	Mixed	Colorectal	PB	440/353	243/226	36/37	NA	NA	NA	Yes
Sakiyama [16] 2005	Japan	Asian	Lung	HB	300/228	500/333	202/124	NA	NA	NA	Yes
Cui [17] 2006	USA	Mixed	Lung	PB	244/468	212/356	41/78	NA	NA	NA	Yes
Cui [17] 2006	USA	Mixed	Multiple	PB	214/474	194/357	35/80	NA	NA	NA	Yes
Zienolddiny [18] 2006	Norway	Caucasian	Lung	HB	NA	NA	NA	195/178	26/21	3/1	Yes
Millikan [19] 2006	USA	Caucasian	Melanoma	PB	731/1513	389/780	73/115	1026/2073	173/360	9/12	Yes
Mechanic [20] 2006	USA	Caucasian	Breast	PB	771/661	409/412	69/60	1049/980	185/150	12/3	Yes
Mechanic [20] 2006	USA	African	Breast	PB	231/231	387/320	139/123	738/642	18/31	1/0	Yes
Huang [21] 2006	USA	Mixed	Colorectal	PB	407/403	243/265	29/29	624/623	78/86	1/7	Yes
García-Closas [22] 2006	Spain	Caucasian	Bladder	HB	629/607	434/445	78/84	885/824	203/182	14/19	Yes
Moreno [23] 2006	Spain	Caucasian	Colorectal	HB	NA	NA	NA	282/257	71/61	7/5	Yes
Shen [24] 2006	USA	Mixed	NHL	PB	260/352	170/169	34/29	NA	NA	NA	Yes
Shen [25] 2006	USA	Mixed	Breast	FB	83/82	63/62	8/7	NA	NA	NA	Yes
Wen [26] 2006	China	Asian	HNC	PB	55/129	81/296	39/100	NA	NA	NA	No
Li [27] 2006	USA	Caucasian	Melanoma	HB	373/370	206/206	23/27	NA	NA	NA	Yes
Wu [28] 2006	USA	Caucasian	Bladder	HB	364/371	225/211	26/18	NA	NA	NA	Yes
Sugimura [29] 2006	Japan	Asian	HNC	HB	20/52	59/112	43/77	NA	NA	NA	Yes
Thirumaran [30] 2006	Multiple	Caucasian	Skin	HB	325/330	172/173	32/30	NA	NA	NA	Yes
Hill [31] 2006	Multiple	Mixed	NHL	PB	599/521	425/331	77/71	NA	NA	NA	Yes
Crew [32] 2007	USA	Mixed	Breast	PB	562/571	371/409	66/71	859/888	156/167	3/10	Yes
Jorgensen [33] 2007	USA	Caucasian	Breast	PB	159/165	93/95	12/15	221/231	37/43	1/1	Yes
Romanowicz [34] 2007	Poland	Caucasian	Breast	NA	NA	NA	NA	31/21	40/48	29/37	Yes
Povey [35] 2007	UK	Caucasian	Melanoma	PB	314/252	169/162	24/27	NA	NA	NA	Yes
Wang [36] 2007	USA	Mixed	Skin	HB	146/200	89/119	11/10	NA	NA	NA	Yes
Shen [37] 2007	Australia	Caucasian	NHL	PB	340/294	170/163	30/27	NA	NA	NA	Yes
McWilliams [38] 2008	USA	Mixed	Pancreatic	HB	NA	NA	NA	411/481	59/111	0/4	Yes
Hooker [39] 2008	USA	African	Prostate	HB	74/100	119/141	61/60	NA	NA	NA	Yes
Smith [40] 2008	USA	Caucasian	Breast	HB	195/256	113/124	12/28	278/358	39/47	7/1	Yes
Smith [40] 2008	USA	African	Breast	HB	13/18	32/37	7/20	51/73	2/2	0/0	Yes
Chang [41] 2008	USA	Hispanic	Lung	HB	60/138	44/127	9/34	97/267	16/31	0/1	Yes
Chang [41] 2008	USA	African	Lung	HB	68/93	119/138	68/49	NA	NA	NA	Yes
Rajaraman [42] 2008	USA	Caucasian	Breast	PB	482/674	288/352	49/53	714/922	124/147		Yes
Frei˘din [43] 2008	Russia	Caucasian	Multiple	HB	38/92	12/36	2/12	NA	NA	NA	No
Hung [44] 2008	Multiple	Mixed	Lung	NA	1852/2485	1155/1510	209/286	2201/2208	306/390	13/21	No for Asp1104His
He [45] 2008	China	Asian	Cervical	HB	35/53	94/80	71/67	NA	NA	NA	No
Pardini [46] 2008	Czech	Caucasian	Colorectal	HB	334/356	177/153	21/23	NA	NA	NA	Yes
Joshi [47] 2009	USA	Caucasian	Colorectal	FB	183/213	125/148		265/313	40/47		NA
El-Zein [48] 2009	USA	Mixed	NHL	HB	104/127	78/80	16/12	NA	NA	NA	Yes
Wen [49] 2009	China	Asian	Bladder	HB	15/45	57/233		NA	NA	NA	NA
Narter [50] 2009	Turkey	Caucasian	Bladder	NA	25/18	28/19	3/3	NA	NA	NA	Yes
Abbasi [51] 2009	German	Caucasian	HNC	PB	137/380	103/230	8/37	203/554	44/90	1/3	Yes
Hussain [52] 2009	China	Asian	Gastric	PB	38/90	104/180	39/91	NA	NA	NA	Yes
McKean-Cowdin [53] 2009	USA	Caucasian	Glioma	PB	499/989	348/657	157/311	NA	NA	NA	No
Pan [54] 2009	USA	Caucasian	esophageal	HB	222/287	145/155	15/15	NA	NA	NA	Yes
Han [55] 2009	USA	Caucasian	Breast	PB	142/285	80/167	17/20	200/401	38/69	0/2	Yes
Liu [56] 2009	USA	Caucasian	Glioma	PB	353/351		20/13	NA	NA	NA	NA
Agalliu [57] 2010	USA	Caucasian	Prostate	PB	NA	NA	NA	1025/1012	183/202	13/5	Yes
Agalliu [57] 2010	USA	African	Prostate	PB	NA	NA	NA	136/78	8/3	0/0	Yes
Rajaraman [58] 2010	USA	Caucasian	Glioma	HB	206/286	123/156	13/26	280/405	56/62	1/4	Yes
Ming-Shiean [59] 2010	China	Asian	Breast	HB	134/159	191/243	76/129	NA	NA	NA	Yes
Li [60] 2010	China	Asian	Liver	HB	174/151	233/265	93/91	NA	NA	NA	Yes
Canbay [61] 2010	Turkey	Caucasian	Gastric	NA	25/148	12/83	3/16	NA	NA	NA	Yes
Figl [62] 2010	Multiple	Caucasian	Melanoma	HB	703/725	409/465	74/84	NA	NA	NA	Yes
Rouissi [63] 2011	Tunis	African	Bladder	HB	95/87	70/86	28/20	NA	NA	NA	Yes
Liu [64] 2011	China	Asian	Colorectal	HB	233/329	603/537	192/219	NA	NA	NA	Yes
Canbay [65] 2011	Turkey	Caucasian	Colorectal	NA	43/148	34/83	2/16	NA	NA	NA	Yes
Gonçalves [66] 2011	Braze	Caucasian	Melanoma	HB	105/109	77/74	10/25	NA	NA	NA	Yes
Ibarrola-Villava [67] 2011	Spain	Caucasian	Melanoma	HB	412/242	222/140	50/24	560/316	117/87	7/3	Yes
Doherty [68] 2011	USA	Mixed	Endometrial	PB	418/408	254/248	42/47	593/620	107/89	3/5	Yes
Biason [69] 2011	Italy	Caucasian	Osteosarcoma	HB	75/141	39/94	16/15	NA	NA	NA	Yes
Krupa [70] 2011	Poland	Caucasian	HNC	HB	NA	NA	NA	221/224	26/29	6/0	Yes
Yu [71] 2011	USA	Caucasian	HNC	HB	NA	NA	NA	837/829	195/209	8/8	Yes
Ma [72] 2012	USA	Caucasian	HNC	HB	648/654	359/350	52/62	NA	NA	NA	Yes
Gil [73] 2012	Poland	Caucasian	Colorectal	HB	86/64	35/31	11/5	119/83	14/15	0/0	Yes
Berhane [74] 2012	India	Asian	Prostate	HB	58/128	72/146	20/26	NA	NA	NA	Yes
Paszkowska-Szczur [75] 2013	Poland	Caucasian	Melanoma	PB	412/869	200/404	28/85	NA	NA	NA	Yes
Wen [80] 2013	China	Asian	Bladder	HB	40/172	46/62	26/44	NA	NA	NA	No
Wang [81] 2013	China	Asian	Glioma	HB	NA	NA	NA	265/609	59/36	6/7	No
Santos [82] 2013	Portugal	Caucasian	HNC	HB	51/106	50/85	4/21	77/168	23/38	2/4	No
Cheng [83] 2013	China	Asian	Glioma	HB	NA	NA	NA	149/182	41/43	17/11	Yes

HNC head and neck cancer, PB population-based study, HB hospital-based study.

### XPG Asp1104His

The evaluations of the association of XPG Asp1104His polymorphism with cancer risk are shown in **Table 2**. Overall, significantly increased risk of cancer was observed in dominant model (OR = 1.05, 95% confidence interval [CI] = 1.00–1.10, *P* value of heterogeneity test [*P_h_*] = 0.001, *I* ^2^ = 40.4) and in Asp/His versus Asp/Asp (OR = 1.06, 95% CI = 1.01–1.11, *P*_h_<0.001, *I* ^2^ = 43.3) when all the eligible studies were pooled into the meta-analysis. Then we performed subgroup analysis by cancer type. No significant association was found in any cancer type, such as breast cancer (dominant model: OR = 1.01, 95% CI = 0.94–1.09, *P*_h_ = 0.128, *I* ^2^ = 33.8, recessive model: OR = 0.95, 95% CI = 0.83–1.09, *P*_h_ = 0.173, *I* ^2^ = 28.6; additive model: OR = 1.00, 95% CI = 0.93–1.09, *P*_h_ = 0.098, *I* ^2^ = 37.8; His/His versus Asp/Asp: OR = 0.99, 95% CI = 0.86–1.14, *P*_h_ = 0.185, *I* ^2^ = 27.2; Asp/His versus Asp/Asp: OR = 1.02, 95% CI = 0.94–1.10, *P*_h_ = 0.136, *I* ^2^ = 32.8), lung cancer (dominant model: OR = 1.13, 95% CI = 0.98–1.31, *P*_h_ = 0.045, *I* ^2^ = 53.4, recessive model: OR = 1.04, 95% CI = 0.93–1.17, *P*_h_ = 0.212, *I* ^2^ = 28.4; additive model: OR = 1.08, 95% CI = 0.98–1.19, *P*_h_ = 0.073, *I* ^2^ = 48.0; His/His versus Asp/Asp: OR = 1.15, 95% CI = 0.94–1.42, *P*_h_ = 0.071, *I* ^2^ = 48.3; Asp/His versus Asp/Asp: OR = 1.13, 95% CI = 0.98–1.31, *P*_h_ = 0.077, *I* ^2^ = 47.3), and so on.

**Table 2:** Stratified analysis of XPG Asp1104His and XPF Arg415Gln polymorphisms on cancer risk.^1^

Genetic model	N	Recessive model		Dominant model		Homozygote		Heterozygote		Additive model	
		OR (95%CI)	*P_h_*/*I^2^* (%)	OR (95%CI)	*P_h_*/*I^2^* (%)	OR (95%CI)	*P_h_*/*I^2^* (%)	OR (95%CI)	*P_h_*/*I^2^* (%)	OR (95%CI)	*P_h_*/*I^2^* (%)
XPG Asp1104His											
Overall	66 (32162/39858)	1.00 (0.94–1.07)*	0.073/21.2	1.05 (1.00–1.10)*	0.001/40.4	1.04 (0.96–1.12)*	0.012/30.9	1.06 (1.01–1.11)*	<0.001/43.3	1.03 (0.99–1.06)*	0.008/32.8
Cancer type											
Bladder cancer	7 (2488/2809)	1.06 (0.72–1.56)*	0.041/56.8	1.10 (0.85–1.44)*	0.001/74.9	1.11 (0.69–1.80)*	0.006/69.7	2	<0.001/77.5	2	<0.001/77.7
Breast cancer	11 (5474/6157)	0.95 (0.83–1.09)	0.173/28.6	1.01 (0.94–1.09)	0.128/33.8	0.99 (0.86–1.14)	0.185/27.2	1.02 (0.94–1.10)	0.136/32.8	1.00 (0.93–1.09)*	0.098/37.8
Colorectal cancer	7 (3471/3638)	0.91 (0.77–1.08)	0.696/0.0	1.07 (0.88–1.29)*	0.004/69.1	1.08 (0.89–1.30)	0.411/0.7	1.11 (0.86–1.42)*	<0.001/78.0	1.03 (0.95–1.12)	0.169/35.7
Glioma	3 (1719/2789)	0.98 (0.81–1.19)	0.262/25.3	1.03 (0.90–1.18)	0.984/0.0	0.97 (0.78–1.19)	0.322/0.0	1.06 (0.92–1.23)	0.810/0.0	1.01 (0.91–1.12)	0.774/0.0
HNC	5 (1709/2691)	0.92 (0.74–1.15)	0.114/46.4	1.01 (0.89–1.16)	0.244/26.6	0.86 (0.67–1.10)	0.257/24.6	1.05 (0.83–1.31)*	0.087/50.8	0.99 (0.90–1.10)	0.735/0.0
NHL	4 (2303/2176)	1.06 (0.84–1.35)	0.389/0.6	1.12 (0.99–1.26)	0.117/49.2	1.11 (0.88–1.42)	0.279/22.0	1.12 (0.99–1.27)	0.194/36.3	1.11 (0.95–1.29)*	0.087/54.4
Lung cancer	7 (5509/6867)	1.04 (0.93–1.17)	0.212/28.4	1.13 (0.98–1.31)*	0.045/53.4	1.15 (0.94–1.42)*	0.071/48.3	1.13 (0.98–1.31)*	0.077/47.3	1.08 (0.98–1.19)*	0.073/48.0
Melanoma	8 (5297/7072)	0.87 (0.69–1.12)*	0.050/50.3	0.97 (0.90–1.04)	0.762/0.0	0.87 (0.68–1.11)*	0.059/48.4	0.98 (0.90–1.06)	0.854/0.0	0.97 (0.91–1.03)	0.336/12.1
Other cancer	14 (4192/5659)	1.07 (0.93–1.22)	0.578/0.0	1.06 (0.97–1.15)	0.406/4.1	1.12 (0.96–1.30)	0.533/0.0	1.05 (0.96–1.15)	0.290/14.9	1.05 (0.98–1.12)	0.675/0.0
XPF Arg415Gln											
Overall	32 (17864/20578)	1.11 (0.81–1.52)*	0.068/30.5	1.04 (0.93–1.15)*	<0.001/62.6	1.10 (0.79–1.54)*	0.035/35.7	1.02 (0.91–1.14)*	<0.001/62.5	1.05 (0.94–1.16)*	<0.001/66.7
Cancer type											
Breast cancer	10 (5086/5542)	1.22 (0.82–1.83)*	0.017/58.9	1.03 (0.92–1.15)	0.167/30.2	1.18 (0.76–1.83)*	0.007/63.8	0.99 (0.87–1.12)	0.277/18.6	1.01 (0.83–1.22)*	0.034/52.0
Lung cancer	3 (2857/3118)	0.75 (0.40–1.41)	0.491/0.0	**0.82 (0.71–0.96)**	0.104/55.7	0.73 (0.39–1.37)	0.466/0.0	**0.83 (0.71–0.97)**	0.132/50.7	0.83 (0.72–0.95)*	0.091/58.4
HNC	4 (1643/2156)	1.47 (0.72–2.98)	0.364/5.8	1.04 (0.88–1.23)	0.359/6.9	1.48 (0.73–3.00)	0.370/4.5	1.02 (0.86–1.21)	0.323/13.9	1.05 (0.90–1.23)	0.302/17.7
Colorectal cancer	4 (1501/1497)	0.51 (0.06–4.35)*	0.069/69.7	0.93 (0.76–1.14)	0.605/0.0	0.51 (0.06–4.45)*	0.067/70.3	0.93 (0.74–1.18)	0.526/0.0	0.90 (0.72–1.11)	0.315/13.4
Glioma	3 (874/1359)	1.51 (0.83–2.74)	0.368/0.0	2	<0.001/87.0	1.61 (0.88–2.93)	0.357/3.0	2	<0.001/88.0	2	0.001/86.0
Other cancer	8 (5903/6906)	1.03 (0.69–1.53)	0.239/24.9	0.95 (0.82–1.10)*	0.048/50.6	1.02 (0.68–1.52)	0.254/23.0	0.95 (0.82–1.11)*	0.040/52.3	0.96 (0.84–1.09)*	0.067/47.0

1 All summary ORs were calculated using fixed-effects models. In the case of significant heterogeneity (indicated by *), ORs were calculated using random-effects models.

2 The results were excluded due to high heterogeneity. The bold values indicate that the results are statistically significant.

We further examined the association of the XPG Asp1104His polymorphism and cancer risk according to cancer type and ethnicity (**Table 3**). For samples of Caucasians, significant association was only be found in head and neck cancer (His/His vs. Asp/His+Asp/Asp: OR = 0.71, 95% CI = 0.51–0.97, *P*_h_ = 0.271, *I* ^2^ = 23.5%) but not bladder cancer (dominant model: OR = 0.99, 95% CI = 0.88–1.12, *P*_h_ = 0.673, *I* ^2^ = 0.0, recessive model: OR = 0.84, 95% CI = 0.50–1.41, *P*_h_ = 0.078, *I* ^2^ = 56.0; additive model: OR = 0.98, 95% CI = 0.89–1.08, *P*_h_ = 0.433, *I* ^2^ = 0.0; His/His versus Asp/Asp: OR = 0.85, 95% CI = 0.51–1.42, *P*_h_ = 0.090, *I* ^2^ = 53.8; Asp/His versus Asp/Asp: OR = 1.01, 95% CI = 0.89–1.15, *P*_h_ = 0.688, *I* ^2^ = 0.0), breast cancer (dominant model: OR = 1.07, 95% CI = 0.92–1.24, *P*_h_ = 0.065, *I* ^2^ = 51.8, recessive model: OR = 1.07, 95% CI = 0.86–1.32, *P*_h_ = 0.221, *I* ^2^ = 28.6; additive model: OR = 1.03, 95% CI = 0.95–1.12, *P*_h_ = 0.113, *I* ^2^ = 43.8; His/His versus Asp/Asp: OR = 1.08, 95% CI = 0.87–1.34, *P*_h_ = 0.215, *I* ^2^ = 29.3; Asp/His versus Asp/Asp: OR = 1.07, 95% CI = 0.91–1.26, *P*_h_ = 0.048, *I* ^2^ = 55.2), and so on. For samples of Asians, significant association was found in lung cancer (dominant model: OR = 1.27, 95% CI = 1.06–1.51, *P*_h_ = 0.133, *I* ^2^ = 50.5%; His/His versus Asp/Asp: OR = 1.28, 95% CI = 1.02–1.60, *P*_h_ = 0.516, *I* ^2^ = 0.0%; additive model: OR = 1.13, 95% CI = 1.02–1.26, *P*_h_ = 0.130, *I* ^2^ = 50.9%).

**Table 3:** Summary ORs (95% CI) categorized by ethnicity for the XPG Asp1104His and XPF Arg415Gln polymorphisms under different genetic models and cancer type.^1^

Ethnicity	Cancer type	N	Recessive model		Dominant model		Homozygote		Heterozygote		Additive model	
			OR (95%CI)	*P_h_*/*I^2^* (%)	OR (95%CI)	*P_h_*/*I^2^* (%)	OR (95%CI)	*P_h_*/*I^2^* (%)	OR (95%CI)	*P_h_*/*I^2^* (%)	OR (95%CI)	*P_h_*/*I^2^* (%)
XPG Asp1104His												
Caucasian	Bladder cancer	4 (2111/2060)	0.84 (0.50–1.41)*	0.078/56.0	0.99 (0.88–1.12)	0.673/0.0	0.85 (0.51–1.42)*	0.090/53.8	1.01 (0.89–1.15)	0.688/0.0	0.98 (0.89–1.08)	0.433/0.0
	Breast cancer	6 (3111/3675)	1.07 (0.86–1.32)	0.221/28.6	1.07 (0.92–1.24)*	0.065/51.8	1.08 (0.87–1.34)	0.215/29.3	1.07 (0.91–1.26)*	0.048/55.2	1.03 (0.95–1.12)	0.113/43.8
	Colorectal cancer	4 (1051/1240)	0.92 (0.57–1.48)	0.262/25.2	1.11 (0.93–1.31)	0.688/0.0	0.97 (0.59–1.58)	0.372/0.0	1.20 (0.96–1.49)	0.397/0.0	1.10 (0.93–1.31)	0.940/0.0
	Glioma	3 (1719/2789)	0.98 (0.81–1.19)	0.262/25.3	1.03 (0.90–1.18)	0.984/0.0	0.97 (0.78–1.19)	0.322/0.0	1.06 (0.92–1.23)	0.810/0.0	1.01 (0.91–1.12)	0.774/0.0
	HNC	3 (1412/1925)	**0.71 (0.51–0.97)**	0.271/23.5	1.04 (0.90–1.20)	0.739/0.0	0.73 (0.53–1.02)	0.378/0.0	1.10 (0.95–1.28)	0.543/0.0	0.98 (0.87–1.10)	0.819/0.0
	Melanoma	8 (5297/7072)	0.87 (0.69–1.12)*	0.050/50.3	0.97 (0.90–1.04)	0.762/0.0	0.87 (0.68–1.11)*	0.059/48.4	0.98 (0.90–1.06)	0.854/0.0	0.97 (0.91–1.03)	0.336/12.1
	Other cancer	5 (1133/1627)	1.21 (0.86–1.70)	0.345/10.7	1.04 (0.89–1.22)	0.599/0.0	1.20 (0.85–1.69)	0.422/0.0	1.02 (0.86–1.20)	0.522/0.0	1.06 (0.93–1.21)	0.501/0.0
Asian	Lung cancer	3 (1428/1105)	1.07 (0.88–1.29)	0.673/0.0	**1.27 (1.06–1.51)**	0.133/50.5	**1.28 (1.02–1.60)**	0.516/0.0	1.35 (0.93–1.96)*	0.073/61.9	**1.13 (1.01–1.26)**	0.559/0.0
	Other cancer	4 (1031/1368)	1.04 (0.85–1.28)	0.350/8.6	1.14 (0.82–1.60)*	0.029/66.9	1.12 (0.88–1.43)	0.176/39.3	1.15 (0.79–1.67)*	0.017/70.7	1.03 (0.92–1.16)	0.187/37.5
XPF Arg415Gln												
Caucasian	Breast cancer	7 (3258/3729)	2.17 (0.68–6.88)*	0.022/61.9	1.10 (0.96–1.25)	0.396/3.9	2.07 (0.56–7.62)*	0.008/68.2	1.05 (0.89–1.23)	0.522/0.0	1.10 (0.89–1.35)*	0.094/46.8
	HNC	4 (1643/2156)	1.47 (0.72–2.98)	0.364/5.8	1.04 (0.88–1.23)	0.359/6.9	1.48 (0.73–3.00)	0.370/4.5	1.02 (0.86–1.21)	0.323/13.9	1.05 (0.90–1.23)	0.302/17.7
	Colorectal cancer	3 (798/781)	1.26 (0.40–4.01)	–	0.99 (0.76–1.30)	0.519/0.0	1.28 (0.40–4.07)	–	0.97 (0.69–1.36)	0.271/17.6	1.00 (0.74–1.36)	0.253/23.5
	Other cancer	4 (4215/5095)	1.20 (0.77–1.87)	0.168/40.6	0.95 (0.85–1.06)	0.549/0.0	1.19 (0.77–1.86)	0.184/38.0	0.94 (0.84–1.05)	0.406/0.0	0.96 (0.87–1.07)	0.666/0.0

1 All summary ORs were calculated using fixed-effects models. In the case of significant heterogeneity (indicated by *), ORs were calculated using random-effects models. The bold values indicate that the results are statistically significant.

We also examined the association of the XPG Asp1104His polymorphism and cancer risk according to cancer type and source of controls (**Table 4**). For the population-based studies, no significant association was found between XPG Asp1104His polymorphism and cancer risk according to cancer type and source of controls. For the hospital-based studies, significant association was observed among breast cancer (recessive model: OR = 0.71, 95% CI = 0.55–0.92, *P*_h_ = 0.262, *I* ^2^ = 24.9%; His/His versus Asp/Asp: OR = 0.74, 95% CI = 0.55–0.98, *P*_h_ = 0.213, *I* ^2^ = 33.3%), colorectal cancer (dominant model: OR = 1.33, 95% CI = 1.15–1.55, *P*_h_ = 0.188, *I* ^2^ = 0.0%; additive model: OR = 1.13, 95% CI = 1.02–1.25, *P*_h_ = 0.971, *I* ^2^ = 0.0%), and other cancer (His/His versus Asp/Asp: OR = 1.22, 95% CI = 1.01–1.47, *P*_h_ = 0.322, *I* ^2^ = 13.5%) but not lung cancer (dominant model: OR = 1.22, 95% CI = 0.91–1.63, *P*_h_ = 0.030, *I* ^2^ = 66.4, recessive model: OR = 1.15, 95% CI = 0.96–1.37, *P*_h_ = 0.105, *I* ^2^ = 51.1; additive model: OR = 1.13, 95% CI = 0.95–1.35, *P*_h_ = 0.057, *I* ^2^ = 60.1; His/His versus Asp/Asp: OR = 1.32, 95% CI = 0.95–1.85, *P*_h_ = 0.095, *I* ^2^ = 53.5; Asp/His versus Asp/Asp: OR = 1.21, 95% CI = 0.89–1.63, *P*_h_ = 0.035, *I* ^2^ = 65.2) and head and neck cancer (dominant model: OR = 1.04, 95% CI = 0.89–1.22, *P*_h_ = 0.548, *I* ^2^ = 0.0, recessive model: OR = 0.88, 95% CI = 0.66–1.16, *P*_h_ = 0.135, *I* ^2^ = 50.1; additive model: OR = 1.00, 95% CI = 0.88–1.13, *P*_h_ = 0.441, *I* ^2^ = 0.0; His/His versus Asp/Asp: OR = 0.90, 95% CI = 0.66–1.22, *P*_h_ = 0.115, *I* ^2^ = 53.2; Asp/His versus Asp/Asp: OR = 1.08, 95% CI = 0.91–1.27, *P*_h_ = 0.591, *I* ^2^ = 0.0), and so on.

**Table 4:** Summary ORs (95% CI) and value of value of the heterogeneity of XPG Asp1104His and XPF Arg415Gln polymorphisms for studies according to source of controls and cancer type^1^.

Source of control	Cancer type	N	Recessive model		Dominant model		Homozygote		Heterozygote		Additive model	
			OR (95%CI)	*P_h_*/*I^2^* (%)	OR (95%CI)	*P_h_*/*I^2^* (%)	OR (95%CI)	*P_h_*/*I^2^* (%)	OR (95%CI)	*P_h_*/*I^2^* (%)	OR (95%CI)	*P_h_*/*I^2^* (%)
XPG Asp1104His												
PB	Breast cancer	6 (4327/4684)	1.06 (0.91–1.24)	0.642/0.0	1.00 (0.92–1.09)	0.130/41.4	1.09 (0.92–1.29)	0.579/0.0	0.99 (0.91–1.08)	0.130/41.3	1.01 (0.95–1.08)	0.130/41.3
	Melanoma	3 (2340/4207)	0.91 (0.58–1.42)*	0.036/70.0	1.00 (0.90–1.11)	0.212/35.5	0.90 (0.56–1.43)	0.372/0.0	1.00 (0.89–1.12)	0.372/0.0	0.97 (0.83–1.13)*	0.073/61.7
	NHL	3 (2105/1957)	1.03 (0.80–1.31)	0.345/6.1	1.11 (0.89–1.38)*	0.062/64.0	1.07 (0.83–1.38)	0.238/30.4	1.11 (0.90–1.37)	0.100/56.7	1.08 (0.90–1.30)*	0.053/66.0
	Other cancer	4 (1709/2395)	0.89 (0.71–1.12)	0.847/0.0	1.08 (0.95–1.23)	0.646/0.0	0.97 (0.76–1.24)	0.900/0.0	1.11 (0.96–1.26)	0.522/0.0	1.02 (0.93–1.13)	0.840/0.0
HB	Bladder cancer	5 (2133/2485)	1.16 (0.92–1.46)	0.219/32.3	2	<0.001/83.2	1.39 (0.86–2.23)*	0.022/68.8	2	<0.001/86.4	2	<0.001/85.5
	Breast cancer	4 (993/1322)	**0.71 (0.55–0.92)**	0.262/24.9	1.06 (0.89–1.26)*	0.100/51.9	**0.74 (0.55–0.98)**	0.213/33.3	1.16 (0.96–1.39)	0.247/27.4	0.97 (0.77–1.22)*	0.039/64.2
	Colorectal cancer	3 (1692/1717)	0.93 (0.76–1.13)	0.525/0.0	**1.33 (1.15–1.55)**	0.188/0.0	1.21 (0.96–1.53)	0.668/0.0	1.29 (0.97–1.72)*	0.072/62.1	**1.13 (1.02–1.25)**	0.971/0.0
	HNC	3 (1286/1519)	0.88 (0.66–1.16)	0.135/50.1	1.04 (0.89–1.22)	0.548/0.0	0.90 (0.66–1.22)	0.115	1.08 (0.91–1.27)	0.591/0.0	1.00 (0.88–1.13)	0.441/0.0
	Lung cancer	4 (1680/1575)	1.15 (0.96–1.37)	0.105/51.1	1.22 (0.91–1.63)*	0.030/66.4	1.32 (0.95–1.85)*	0.092/53.5	1.21 (0.89–1.63)*	0.035/65.2	1.13 (0.95–1.35)*	0.057/60.1
	Melanoma	5 (2957/2865)	0.88 (0.70–1.09)	0.145/41.5	0.94 (0.85–1.04)	0.981/0.0	0.86 (0.69–1.08)	0.213/31.3	0.95 (0.85–1.06)	0.915/0.0	0.94 (0.86–1.02)	0.766/0.0
	Other cancer	9 (2443/3017)	1.18 (0.99–1.41)	0.576/0.0	1.05 (0.94–1.18)	0.171/31.0	**1.22 (1.01–1.47)**	0.322/13.5	1.02 (0.90–1.15)	0.155/32.9	1.07 (0.98–1.16)	0.361/8.9
XPF Arg415Gln												
PB	Breast cancer	6 (4356/4687)	1.05 (0.29–3.77)*	0.098/49.0	1.02 (0.90–1.16)	0.158/37.3	1.05 (0.29–3.81)*	0.093/49.7	1.00 (0.87–1.15)	0.133/43.2	0.96 (0.77–1.20)*	0.069/54.0
	Other cancer	5 (3647/4879)	1.48 (0.84–2.60)	0.354/7.9	1.03 (0.91–1.17)	0.477/0.0	1.48 (0.84–2.60)	0.386/1.2	1.02 (0.90–1.15)	0.286/20.2	1.05 (0.93–1.17)	0.731/0.0
HB	Breast cancer	4 (730/855)	3.66 (0.38–34.9)*	0.009/78.7	1.04 (0.78–1.39)	0.178/38.9	3.39 (0.26–43.9)*	0.003/82.8	0.92 (0.68–1.25)	0.463/0.0	1.13 (0.73–1.73)*	0.054/60.7
	Other cancer	3 (2256/2027)	0.70 (0.39–1.25)	0.341/6.9	0.79 (0.59–1.07)*	0.035/70.1	0.69 (0.38–1.24)	0.347/5.6	0.81 (0.59–1.10)*	0.033/70.8	0.80 (0.61–1.05)*	0.045/67.7

1 All summary ORs were calculated using fixed-effects models. In the case of significant heterogeneity (indicated by *), ORs were calculated using random-effects models.

2 The results were excluded due to high heterogeneity. The bold values indicate that the results are statistically significant. PB Population-based studies, HB Hospital-based studies, the bold values indicate that the results are statistically significant.

There was significant heterogeneity among these studies for dominant model comparison (*P*_h_ = 0.001), recessive model comparison (*P*_h_ = 0.073), additive model comparison (*P*_h_ = 0.008), homozygote model comparison (*P*_h_ = 0.012), and heterozygote model comparison (*P*_h_<0.001). Then, we assessed the source of heterogeneity by ethnicity, cancer type, source of controls, HWE, and sample size. The results indicated that sample size (recessive model: *P* = 0.038) but not cancer type (dominant model: *P* = 0.782; recessive model: *P* = 0.208; His/His versus Asp/Asp: *P* = 0.336; Asp/His versus Asp/Asp: *P* = 0.825; additive model: *P* = 0.556), ethnicity (dominant model: *P* = 0.298; recessive model: *P* = 0.119; His/His versus Asp/Asp: *P* = 0.066; Asp/His versus Asp/Asp: *P* = 0.449; additive model: *P* = 0.241), source of controls (dominant model: *P* = 0.433; recessive model: *P* = 0.821; His/His versus Asp/Asp: *P* = 0.634; Asp/His versus Asp/Asp: *P* = 0.358; additive model: *P* = 0.429), and HWE (dominant model: *P* = 0.126; recessive model: *P* = 0.660; His/His versus Asp/Asp: *P* = 0.272; Asp/His versus Asp/Asp: *P* = 0.123; additive model: *P* = 0.217) contributed to substantial heterogeneity among the meta-analysis. Examining genotype frequencies in the controls, significant deviation from HWE was detected in the eight studies [10], [26], [43], [44], [45], [53], [80], [81]. When these studies were excluded, the results were changed among overall cancer (dominant model: OR = 1.03, 95% CI = 0.99–1.08), Asians of lung cancer (dominant model: OR = 1.15, 95% CI = 0.95–1.41; His/His versus Asp/Asp: OR = 1.20, 95% CI = 0.92–1.55; additive model: OR = 1.10, 95% CI = 0.96–1.25), and hospital-based studies of other cancer (recessive model: OR = 1.23, 95% CI = 1.02–1.49; His/His versus Asp/Asp: OR = 1.20, 95% CI = 0.97–1.48), as shown in **Table 5**. In addition, when the meta-analysis was performed excluding studies with small sample sizes, the results did not change among overall cancer studies and any subgroup analysis, as shown in **Table 6**. Last, a single study involved in the meta–analysis was deleted each time to reflect the influence of individual data set to the pooled ORs, the results were changed among Caucasians of head and neck cancer (recessive model: OR = 0.75, 95% CI = 0.53–1.06), hospital-based studies of breast cancer (recessive model: OR = 1.22, 95% CI = 0.98–1.52; Gln/Gln versus Arg/Arg: OR = 0.79, 95% CI = 0.51–1.24), hospital-based studies of colorectal cancer (dominant model: OR = 1.15, 95% CI = 0.92–1.45; additive model: OR = 1.12, 95% CI = 0.92–1.35).

**Table 5:** Summary ORs (95% CI) and value of the heterogeneity of XPG Asp1104His and XPF Arg415Gln polymorphisms under different genetic models according to studies with HWE on cancer risk.^1^

Genetic model	No. comparisons (SZ case/control)	Recessive model		Dominant model		Homozygote		Heterozygote		Additive model	
		OR (95%CI)	*P_h_/I^2^* (%)	OR (95%CI)	*P_h_/I^2^* (%)	OR (95%CI)	*P_h_/I^2^* (%)	OR (95%CI)	*P_h_/I^2^* (%)	OR (95%CI)	*P_h_/I^2^* (%)
XPG Asp1104His											
Overall	58 (26988/31954)	0.99 (0.92–1.07)*	0.068/22.9	1.03 (0.99–1.08)*	0.092/20.6	1.02 (0.94–1.11)*	0.066/23.4	1.04 (1.00–1.09)*	0.055/24.5	1.02 (0.99–1.05)	0.139/17.3
Cancer type											
Bladder cancer	6 (2376/2531)	0.95 (0.62–1.47)*	0.065/54.9	0.97 (0.87–1.09)	0.724/0.0	0.94 (0.73–1.20)	0.112/46.6	0.98 (0.87–1.11)	0.517/0.0	0.98 (0.89–1.08)	0.599/0.0
Glioma	2 (715/832)	0.99 (0.61–1.60)	0.102/62.6	1.04 (0.78–1.38)	–	0.69 (0.35–1.38)	–	1.09 (0.81–1.47)	–	0.97 (0.77–1.24)	–
HNC	3 (1429/1954)	0.88 (0.67–1.16)	0.240/29.9	1.06 (0.92–1.23)	0.454/0.0	0.90 (0.67–1.22)	0.194/39.0	1.10 (0.95–1.28)	0.462/0.0	1.02 (0.91–1.14)	0.537/0.0
Lung cancer	5 (1983/2275)	1.12 (0.95–1.34)	0.139/42.4	1.12 (0.98–1.28)	0.348/10.2	1.19 (0.98–1.44)	0.117/45.8	1.11 (0.96–1.27)	0.694/0.0	1.08 (0.94–1.24)*	0.098/48.9
Other cancer	12 (3940/5319)	1.08 (0.93–1.24)	0.532/0.0	1.05 (0.96–1.14)	0.665/0.0	1.10 (0.94–1.29)	0.667/0.0	1.04 (0.95–1.14)	0.459/0.0	1.05 (0.98–1.12)	0.835/0.0
Ethnicity and cancer type											
Lung cancer/Asian	2 (1118/794)	1.10 (0.88–1.38)	0.463/0.0	1.15 (0.95–1.41)	0.710/0.0	1.20 (0.92–1.55)	0.517/0.0	1.14 (0.92–1.40)	0.894/0.0	1.10 (0.96–1.25)	0.484/0.0
Other cancer/Caucasian	4 (1081/1487)	1.30 (0.92–1.85)	0.473/0.0	1.07 (0.90–1.26)	0.679/0.0	1.29 (0.91–1.85)	0.618/0.0	1.03 (0.87–1.23)	0.418/0.0	1.09 (0.95–1.25)	0.811/0.0
Other cancer/Asian	3 (831/1168)	1.03 (0.81–1.30)	0.199/38.1	0.96 (0.70–1.17)	0.109/54.8	1.02 (0.78–1.34)	0.240/30.0	1.01 (0.71–1.44)*	0.071/62.1	0.99 (0.87–1.13)	0.269/23.8
Source of controls and cancer type											
Bladder cancer/HB	4 (2021/2207)	1.08 (0.84–1.40)	0.254/27.1	0.97 (0.85–1.10)	0.425/0.0	1.04 (0.80–1.36)	0.299/17.2	0.96 (0.84–1.10)	0.296/17.9	1.00 (0.90–1.10)	0.352/4.1
Lung cancer/HB	3 (1370/1264)	1.20 (0.80–1.79)	0.077/61.0	1.13 (0.96–1.34)	0.112/54.3	1.23 (0.76–2.00)*	0.050/66.5	1.09 (0.91–1.30)	0.347/5.5	1.09 (0.85–1.40)*	0.029/71.8
Other cancer/HB	7 (2191/2677)	**1.23 (1.02–1.49)**	0.595/0.0	1.03 (0.92–1.16)	0.375/7.0	1.20 (0.97–1.48)	0.394/4.3	0.99 (0.87–1.12)	0.324/13.9	1.07 (0.97–1.17)	0.515/0.0
XPF Arg415Gln											
Overall	30 (17432/19716)	1.09 (0.78–1.54)*	0.047/34.6	0.99 (0.91–1.07)*	0.026/36.4	1.07 (0.74–1.53)*	0.027/38.6	0.97 (0.89–1.05)*	0.059/31.4	1.00 (0.91–1.08)	0.003/47.8
Cancer type											
Glioma	2 (544/707)	1.44 (0.71–2.93)	0.161/49.2	1.28 (0.96–1.70)	0.868/0.0	1.49 (0.73–3.03)	0.163/48.5	1.25 (0.92–1.69)	0.716/0.0	1.28 (0.99–1.65)	0.525/0.0
HNC	3 (1541/1946)	1.58 (0.72–3.46)	0.204/37.1	1.02 (0.85–1.21)	0.277/22.1	1.57 (0.72–3.45)	0.206/36.6	0.99 (0.83–1.19)	0.264/25.0	1.04 (0.88–1.22)	0.201/37.7

1 All summary ORs were calculated using fixed-effects models. In the case of significant heterogeneity (indicated by *), ORs were calculated using random-effects models. The bold values indicate that the results are statistically significant.

**Table 6:** Summary ORs (95% CI) and value of the heterogeneity of XPG Asp1104His and XPF Arg415Gln polymorphisms under different genetic models according to studies with a minimum of 200 participants on cancer risk.^1^

Genetic model	No. comparisons (SZ case/control)	Recessive model		Dominant model		Homozygote		Heterozygote		Additive model	
		OR (95%CI)	*P_h_/I^2^* (%)	OR (95%CI)	*P_h_/I^2^* (%)	OR (95%CI)	*P_h_/I^2^* (%)	OR (95%CI)	*P_h_/I^2^* (%)	OR (95%CI)	*P_h_/I^2^* (%)
XPG Asp1104His											
Overall	63 (32002/39603)	1.01 (0.94–1.07)*	0.085/20.6	1.05 (1.01–1.10)*	<0.001/42.5	1.04 (0.97–1.13)*	0.012/31.6	1.06 (1.01–1.11)*	<0.001/45.8	1.03 (0.99–1.06)*	0.007/33.5
Cancer type											
Breast cancer	10 (5422/6082)	0.97 (0.85–1.11)	0.265/19.3	1.03 (0.93–1.14)*	0.089/40.3	1.00 (0.87–1.15)	0.205/25.9	1.04 (0.93–1.16)*	0.098/39.0	1.01 (0.93–1.09)*	0.096/39.3
Bladder cancer	6 (2432/2769)	1.08 (0.71–1.63)	0.023/64.7	2	<0.001/79.0	1.14 (0.68–1.91)*	0.003/75.4	2	<0.001/82.0	2	<0.001/82.1
Other cancer	13 (4140/5519)	1.08 (0.94–1.24)	0.618/0.0	1.07 (0.98–1.16)	0.425/2.1	1.13 (0.97–1.32)	0.596/0.0	1.06 (0.96–1.15)	0.252/18.9	1.06 (0.99–1.13)	0.783/0.0
XPF Arg415Gln											
Overall	31 (17811/20503)	1.11 (0.81–1.52)*	0.068/30.5	1.04 (0.93–1.15)*	<0.001/63.7	1.10 (0.79–1.54)*	0.035/35.7	1.02 (0.91–1.14)*	<0.001/63.7	1.05 (0.94–1.16)*	<0.001/67.8
Cancer type											
Breast cancer	9 (5033/5467)	1.54 (0.59–3.99)*	0.017/58.9	1.02 (0.91–1.15)	0.119/37.5	1.49 (0.52–4.25)	0.007/63.8	0.98 (0.87–1.12)	0.207/27.8	1.00 (0.83–1.22)*	0.021/57.7

1 All summary ORs were calculated using fixed-effects models. In the case of significant heterogeneity (indicated by *), ORs were calculated using random-effects models.

2 The results were excluded due to high heterogeneity. The bold values indicate that the results are statistically significant.

**Figure 2:**
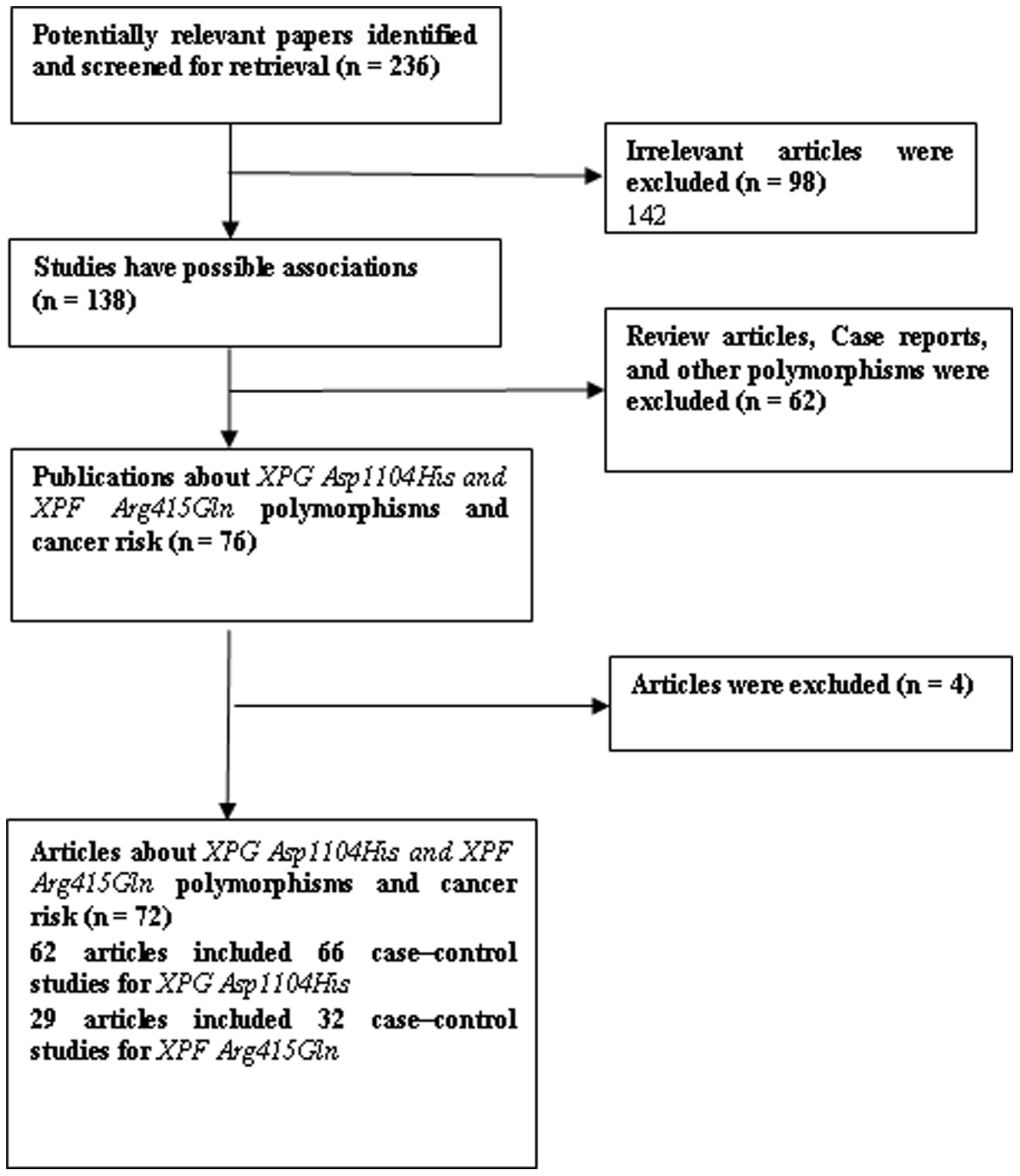
Begg's funnel plot for publication bias test between XPG Asp1104His polymorphism and cancer risk (additive model and dominant model).

Both Begg's funnel plot and Egger's test were performed to assess the publication bias of literatures. The Egger's test results (dominant model: *P* = 0.245; recessive model: *P* = 0.482; additive model: *P* = 0.581; Homozygote model: *P* = 0.443; Heterozygote model: *P* = 0.148) and Begg's funnel plot (**Fig. 2**) suggested no evidence of publication bias in the meta-analysis.

### XPF Arg415Gln

The evaluations of the association of XPF Arg415Gln polymorphism with cancer risk are shown in **Table 2**. No significant association was observed between XPF Arg415Gln polymorphism and cancer risk when all the eligible studies were pooled into the meta-analysis (dominant model: OR = 1.04, 95% CI = 0.93–1.15, *P*_h_<0.001, *I* ^2^ = 62.6; recessive model: OR = 1.11, 95% CI = 0.81–1.52, *P*_h_ = 0.068, *I* ^2^ = 30.5; additive model: OR = 1.05, 95% CI = 0.94–1.16, *P*_h_<0.001, *I* ^2^ = 66.7; Gln/Gln versus Arg/Arg: OR = 1.10, 95% CI = 0.79–1.54, *P*_h_ = 0.035, *I* ^2^ = 35.7; Arg/Gln versus Arg/Arg: OR = 1.02, 95% CI = 0.91–1.14, *P*_h_<0.001, *I* ^2^ = 62.5). Then we performed subgroup analysis by cancer type. Significant association was found among lung cancer (dominant model: OR = 0.82, 95% CI = 0.71–0.96, *P*_h_ = 0.104, *I* ^2^ = 55.7%; Arg/Gln versus Arg/Arg: OR = 0.83, 95% CI = 0.71–0.97, *P*_h_ = 0.132, *I* ^2^ = 50.7%; additive model: OR = 0.83, 95% CI = 0.72–0.95, *P*_h_ = 0.091, *I* ^2^ = 58.4%) but not breast cancer (dominant model: OR = 1.03, 95% CI = 0.92–1.15, *P*_h_ = 0.167, *I* ^2^ = 30.2; recessive model: OR = 1.22, 95% CI = 0.82–1.83, *P*_h_ = 0.017, *I* ^2^ = 58.9; additive model: OR = 1.01, 95% CI = 0.83–1.22, *P*_h_ = 0.034, *I* ^2^ = 52.0; Gln/Gln versus Arg/Arg: OR = 1.18, 95% CI = 0.76–1.83, *P*_h_ = 0.007, *I* ^2^ = 63.8; Arg/Gln versus Arg/Arg: OR = 0.99, 95% CI = 0.87–1.12, *P*_h_ = 0.277, *I* ^2^ = 18.6), head and neck cancer (dominant model: OR = 1.04, 95% CI = 0.88–1.23, *P*_h_ = 0.359, *I* ^2^ = 6.9; recessive model: OR = 1.47, 95% CI = 0.72–2.98, *P*_h_ = 0.364, *I* ^2^ = 5.8; additive model: OR = 1.05, 95% CI = 0.90–1.23, *P*_h_ = 0.302, *I* ^2^ = 17.7; Gln/Gln versus Arg/Arg: OR = 1.48, 95% CI = 0.73–3.00, *P*_h_ = 0.370, *I* ^2^ = 4.5; Arg/Gln versus Arg/Arg: OR = 1.02, 95% CI = 0.86–1.21, *P*_h_ = 0.323, *I* ^2^ = 13.9), and so on.

We further examined the association of the XPF Arg415Gln polymorphism and cancer risk according to cancer type and ethnicity (**Table 3**). For the samples of Caucasians, no significant association was found among breast cancer (dominant model: OR = 1.10, 95% CI = 0.96–1.25, *P*_h_ = 0.396, *I* ^2^ = 3.9; recessive model: OR = 2.17, 95% CI = 0.68–6.88, *P*_h_ = 0.022, *I* ^2^ = 61.9; additive model: OR = 1.10, 95% CI = 0.89–1.35, *P*_h_ = 0.094, *I* ^2^ = 46.8; Gln/Gln versus Arg/Arg: OR = 2.07, 95% CI = 0.56–7.62, *P*_h_ = 0.008, *I* ^2^ = 68.2; Arg/Gln versus Arg/Arg: OR = 1.05, 95% CI = 0.89–1.23, *P*_h_ = 0.522, *I* ^2^ = 0.0), head and neck cancer (dominant model: OR = 1.04, 95% CI = 0.88–1.23, *P*_h_ = 0.359, *I* ^2^ = 6.9; recessive model: OR = 1.47, 95% CI = 0.72–2.98, *P*_h_ = 0.364, *I* ^2^ = 5.8; additive model: OR = 1.05, 95% CI = 0.90–1.23, *P*_h_ = 0.302, *I* ^2^ = 17.7; Gln/Gln versus Arg/Arg: OR = 1.48, 95% CI = 0.73–3.00, *P*_h_ = 0.370, *I* ^2^ = 4.5; Arg/Gln versus Arg/Arg: OR = 1.02, 95% CI = 0.86–1.21, *P*_h_ = 0.323, *I* ^2^ = 13.9), and so on.

We also examined the association of the XPF Arg415Gln polymorphism and cancer risk according to cancer type and source of controls (**Table 4**). For the population-based studies, no significant association was found among breast cancer (dominant model: OR = 1.02, 95% CI = 0.90–1.16, *P*_h_ = 0.158, *I* ^2^ = 37.3; recessive model: OR = 1.05, 95% CI = 0.29–3.77, *P*_h_ = 0.098, *I* ^2^ = 49.0; additive model: OR = 0.96, 95% CI = 0.77–1.20, *P*_h_ = 0.069, *I* ^2^ = 54.0; Gln/Gln versus Arg/Arg: OR = 1.05, 95% CI = 0.29–3.81, *P*_h_ = 0.093, *I* ^2^ = 49.7; Arg/Gln versus Arg/Arg: OR = 1.00, 95% CI = 0.87–1.15, *P*_h_ = 0.133, *I* ^2^ = 43.2) and other cancer (dominant model: OR = 1.03, 95% CI = 0.91–1.17, *P*_h_ = 0.477, *I* ^2^ = 0.0; recessive model: OR = 1.48, 95% CI = 0.84–2.60, *P*_h = _0.354, *I* ^2^ = 7.9; additive model: OR = 1.05, 95% CI = 0.93–1.17, *P*_h = _0.731, *I* ^2^ = 0.0; Gln/Gln versus Arg/Arg: OR = 1.48, 95% CI = 0.84–2.60, *P*_h = _0.386, *I* ^2^ = 1.2; Arg/Gln versus Arg/Arg: OR = 1.02, 95% CI = 0.90–1.15, *P*_h = _0.286, *I* ^2^ = 20.2). For the hospital-based studies, no significant association was also observed among breast cancer (dominant model: OR = 1.04, 95% CI = 0.78–1.39, *P*_h = _0.178, *I* ^2^ = 38.9; recessive model: OR = 3.66, 95% CI = 0.38–34.9, *P*_h = _0.009, *I* ^2^ = 78.7; additive model: OR = 1.13, 95% CI = 0.73–1.73, *P*_h = _0.054, *I* ^2^ = 60.7; Gln/Gln versus Arg/Arg: OR = 3.39, 95% CI = 0.26–43.9, *P*_h = _0.003, *I* ^2^ = 82.8; Arg/Gln versus Arg/Arg: OR = 0.92, 95% CI = 0.68–1.25, *P*_h = _0.463, *I* ^2^ = 0.0) and other cancer (dominant model: OR = 0.79, 95% CI = 0.59–1.07, *P*_h = _0.035, *I* ^2^ = 70.1; recessive model: OR = 0.70, 95% CI = 0.39–1.25, *P*_h = _0.341, *I* ^2^ = 6.9; additive model: OR = 0.80, 95% CI = 0.61–1.05, *P*_h = _0.045, *I* ^2^ = 67.7; Gln/Gln versus Arg/Arg: OR = 0.69, 95% CI = 0.38–1.24, *P*_h = _0.347, *I* ^2^ = 5.6; Arg/Gln versus Arg/Arg: OR = 0.81, 95% CI = 0.59–1.10, *P*_h = _0.033, *I* ^2^ = 70.8).

There was significant heterogeneity among these studies for dominant model comparison (*P*_h_<0.001), recessive model comparison (*P*_h_ = 0.068), additive model comparison (*P*_h_<0.001), homozygote model comparison (*P*_h_ = 0.035), and heterozygote model comparison (*P*_h_<0.001). Then, we assessed the source of heterogeneity by ethnicity, cancer type, source of controls, HWE, and sample size. Meta-regression analysis indicated that HWE (Arg/Gln versus Arg/Arg: *P*<0.001; additive model: *P* = 0.001; dominant model: *P*<0.001) and ethnicity (Gln/Gln versus Arg/Arg: *P* = 0.001; recessive model: *P* = 0.001) but not cancer type (dominant model: *P* = 0.446; recessive model: *P* = 0.344; Gln/Gln versus Arg/Arg: *P* = 0.314; Arg/Gln versus Arg/Arg: *P* = 0.694; additive model: *P* = 0.456), source of controls (dominant model: *P* = 0.710; recessive model: *P* = 0.218; Gln/Gln versus Arg/Arg: *P* = 0.221; Arg/Gln versus Arg/Arg: *P* = 0.558; additive model: *P* = 0.962), and sample size (dominant model: *P* = 0.125; recessive model: *P* = 0.255; Gln/Gln versus Arg/Arg: *P* = 0.076; Arg/Gln versus Arg/Arg: *P* = 0.252; additive model: *P* = 0.153) contributed to substantial heterogeneity among the meta-analysis. Examining genotype frequencies in the controls, significant deviation from HWE was detected in the two studies [81], [82]. When these two studies were excluded, the results were not changed among overall cancer and any subgroup analysis, as shown in **Table 5**. In addition, when the meta-analysis was performed excluding studies with small sample sizes, the results did not also change among overall cancer and any subgroup analysis, as shown in **Table 6**. Last, a single study involved in the meta–analysis was deleted each time to reflect the influence of individual data set to the pooled ORs, the results did not also change among this meta-analysis, indicating that our results did not influenced statistically robust.

**Figure 3:**
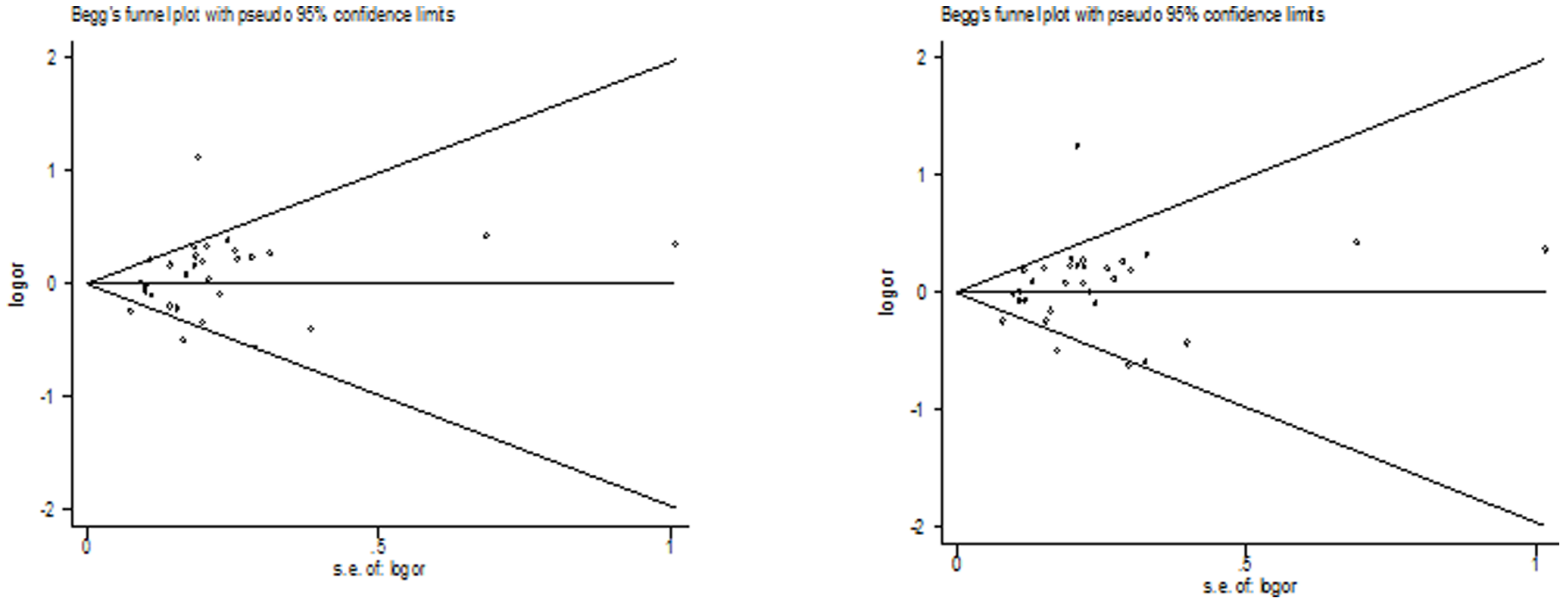
Begg's funnel plot for publication bias test between XPF Arg415Gln polymorphism and cancer risk (additive model and dominant model).

Both Begg's funnel plot and Egger's test were performed to assess the publication bias of literatures. The Egger's test results (*P* = 0.171; recessive model: *P* = 0.437; additive model: *P* = 0.114; Homozygote model: *P* = 0.425; Heterozygote model: *P* = 0.229) and Begg's funnel plot (**Fig. 3**) suggested no evidence of publication bias in the meta-analysis.

## Discussion

NER is a crucial DNA repair mechanism, which counteracts the consequences of mutagenic exposure of cell. XPF and XPG are both central players in the NER pathway, and involved in incision 5′ and 3′-ends, respectively, of the DNA lesion. A number of epidemiological studies have evaluated the association between XPG Asp1104His and XPF Arg415Gln polymorphisms and cancer risk, but the results remain inconclusive.

For instance, McWilliams et al. [38] reported a significantly decreased pancreatic cancer risk with XPF Arg415Gln polymorphism (*P* = 0.003). But Liu et al. [64] reported a significantly increased colorectal cancer risk associated with the variant allele of XPG Asp1104His. Goncalves et al. [66] found that significantly decreased melanoma cancer risk with the XPG 1104 His/His genotype (OR = 0.32; 95% CI = 0.13–0.75). However, Berhane et al. [74] found that statistically significant increased risk of prostate cancer was observed on individuals that posses His/His genotype of XPG (OR = 2.53, 95% CI = 0.99–6.56, *P* = 0.031). Ming-Shiean et al. [59] reported a significantly increased breast cancer risk with the variant allele of XPG Asp1104His (OR = 1.42; 95% CI = 1.08–1.97). He et al. [45] found that Women carrying homozygous Asp1104Asp genotypes had a significantly decreased risk of cervical or cervical squamous cell carcinoma compared to His1104Asp or His1104His genotypes. Smith et al. [8] reported a statistically significant difference in the XPF Arg415Gln genotype distributions between breast cancer cases and controls (*P* = 0.02). Furthermore, Kumar et al. [9] reported a marginally significant increase in breast cancer risk associated with the variant allele of XPG Asp1104His. What's more, more studies did not find obvious association among them. In order to resolve this conflict, a meta-analysis of 98 eligible studies including 32,162 cases and 39,858 controls for XPG Asp1104His and 17,864 cases and 20,578 controls for XPF Arg415Gln was performed to derive a more precise estimation of the association.

Overall, significantly elevated cancer risk was found when all studies were pooled into the meta-analysis of XPG Asp1104His (dominant model: OR = 1.05, 95% CI = 1.00–1.10; Asp/His versus Asp/His: OR = 1.06, 95% CI = 1.01–1.11). Based on biochemical properties described for XPG Asp1104His and XPF Arg415Gln polymorphisms, we would expect that the His or Gln alleles would be associated for all types of cancer. However, our results showed that such association was observed just among lung cancer (dominant model: OR = 0.82, 95% CI = 0.71–0.96; Asp/His versus Asp/Asp: OR = 0.83, 95% CI = 0.71–0.97; additive model: OR = 0.83, 95% CI = 0.72–0.95) for XPF Arg415Gln and hospital-based studies of other cancer (dominant model: OR = 1.23, 95% CI = 1.02–1.49) for XPG Asp1104His, suggesting that other factors may be modulating the XPG Asp1104His and XPF Arg415Gln polymorphisms functionality. However, the exact mechanism for association between different tumor sites and XPG Asp1104His and XPF Arg415Gln polymorphisms was not clear, carcinogenetic mechanism may differ by different tumor sites and the XPG Asp1104His and XPF Arg415Gln genetic variants may exert varying effects in different cancers. Hung et al. [44] reported a marginally significantly decreased lung cancer risk with the variant allele of XPF Arg415Gln (dominant model: OR = 0.78, 95% CI = 0.67–0.91). Our results seem to confirm and establish the trend in the meta-analysis of XPF Arg415Gln polymorphism and lung cancer risk that the data by Hung et al. [40] had indicated. However, at any case, the association between XPF Arg415Gln and lung cancer risk remain an open field, as the number of studies (n = 3 for Arg415Gln) is considerably smaller than that needed for the achievement of robust conclusions [94]. In the subgroup analysis by source of control and cancer type, significantly increased other cancer association was found among the hospital-based studies for the XPG Asp1104His polymorphism, but not the population-based studies. However, the hospital-based studies may have certain biases for such controls and may only represent a sample of an ill-defined reference population, and may not be representative of the general population or it may be that numerous subjects in the population-based controls were susceptible individuals. The results only indicate that participation of XPG Asp1104His may be a genetic susceptibility for other cancer. Therefore, the use of proper and representative population-based controls control subjects is important to reduce biases and in such genetic studies.

We noticed with great interest that 2 previous meta-analysis had been reported on the cancer risk with XPG Asp1104His and XPF Arg415Gln polymorphisms [84], [85]. Zhu et al. [84] had 49 case–control studies, in which a total of 23,490 cases and 27,168 controls were included. Their meta-analysis suggested that it was unlikely that the XPG Asp1104His polymorphism may contribute to individual susceptibility to cancer risk. Shi et al. [85] had 23 case-control studies, in which a total of 14,632 cancer cases and 15,545 controls. Their meta-analysis suggested that it was unlikely that the XPF Arg415Gln polymorphism may contribute to individual susceptibility to cancer risk. However, several published studies were not included in that meta-analysis [84], [85]. By analyzing a larger number of studies than the previous meta-analysis [84], [85], our meta-analysis included 32,162 cases and 39,858 controls (from 66 studies) for XPG Asp1104His and 17,864 cases and 20,578 controls (from 32 studies) for XPF Arg415Gln to perform the two gene polymorphisms and cancer risk. Our meta-analysis suggests that XPF Arg415Gln polymorphism may be associated with decreased lung cancer risk and XPG Asp1104His may be a low-penetrant risk factor in some cancer development. Our results seem to confirm and establish the trend in the meta-analysis of the XPG Asp1104His and XPF Arg415Gln polymorphisms according to the previous meta-analysis [84], [85].

In the present meta-analysis, between-studies heterogeneity was observed between XPG Asp1104His and XPF Arg415Gln polymorphisms and cancer of risk. Meta-regression analysis indicated that HWE contributed to substantial heterogeneity among the meta-analysis for XPF Arg415Gln polymorphism and sample size contributed to substantial heterogeneity among the meta-analysis for XPG Asp1104His. Deviation of HWE may reflect methodological problems such as genotyping errors, population stratification or selection bias. When these studies were excluded, the results were changed among overall cancer and some subgroup analyses for XPG Asp1104His, indicating that our meta-analysis was not statistically robust. Hence, significant association may be not existed in some cancer types when the results were changed. When the meta-analysis was performed excluding studies with small sample sizes, the results did not change among overall cancer studies and any subgroup analysis, indicating that small sample sizes did not influenced statistically robust.

Our meta-analysis has several strengths. First, a systematic review of the association of XPG Asp1104His and XPF Arg415Gln polymorphisms with cancer risk is statistically more powerful than any single study. Second, the quality of eligible studies included in current meta-analysis was satisfactory and met our inclusion criterion. Third, we did not detect any publication bias indicating that the whole pooled results should be unbiased. However, although we have put considerable efforts and resources into testing possible association between XPG Asp1104His and XPF Arg415Gln polymorphisms and cancer risk, there are still some limitations inherited from the published studies. First, our results were based on single-factor estimations without adjustment for other risk factors including alcohol usage, environmental factors and other lifestyles. At lower levels of alcohol consumption, the difference in cancer risk between the various gene carriers was less striking. And higher levels of alcohol consumption result in production of more acetaldehyde which then can exert its carcinogenic effect [95]. Second, in the subgroup analysis may have had insufficient statistical power to check an association. Third, the controls were not uniformly defined. Some studies used a healthy population as the reference group, whereas others selected hospital patients without organic cancer as the reference group. Therefore, non-differential misclassification bias is possible because these studies may have included the control groups who have different risks of developing cancer of various organs.

In conclusion, this meta-analysis suggests that XPF Arg415Gln polymorphism may be associated with decreased lung cancer risk and XPG Asp1104His may be a low-penetrant risk factor in some cancer development. However, it is necessary to conduct large sample studies using standardized unbiased genotyping methods, homogeneous cancer patients and well-matched controls. Moreover, further studies estimating the effect of gene–gene and gene–environment interactions may eventually lead to our better, comprehensive understanding of the association between the XPF Arg415Gln and XPG Asp1104His polymorphisms and cancer risk.

## Supporting Information

Checklist S1 **PRISMA Checklist.**
(DOC)

## References

[1] Wood RD, Mitchell M, Sgouros J, Lindahl T (2001) Human DNA repair genes. Science 291: 1284–1289. 11181991 10.1126/science.1056154

[2] Friedberg EC (2001) How nucleotide excision repair protects against cancer. Nature Rev Cancer 1: 22–33. 11900249 10.1038/35094000

[3] Cleaver JE (2000) Common pathways for ultraviolet skin carcinogenesis in the repair and replication defective groups of xeroderma pigmentosum. J Dermatol Sci 23: 1–11. 10699759 10.1016/s0923-1811(99)00088-2

[4] O'Donovan A, Davies AA, Moggs JG, West SC, Wood RD (1994) XPG endonuclease makes the 30 incision in human DNA nucleotide excision repair. Nature 371: 432–435. 8090225 10.1038/371432a0

[5] Wakasugi M, Reardon JT, Sancar A (1997) The non-catalytic function of XPG protein during dual incision in human nucleotide excision repair. J Biol Chem 272: 16030–16034. 9188507 10.1074/jbc.272.25.16030

[6] Araujo SJ, Nigg EA, Wood RD (2001) Strong functional interactions of TFIIH with XPC and XPG in human DNA nucleotide excision repair, without a preassembled repairosome. Mol Cell Biol 21: 2281–2291. 11259578 10.1128/MCB.21.7.2281-2291.2001 PMC86862

[7] Gillet LC, Scharer OD (2006) Molecular mechanisms of mammalian global genome nucleotide excision repair. Chem Rev 106: 253–276. 16464005 10.1021/cr040483f

[8] Smith TR, Levine EA, Perrier ND, Miller MS, Freimanis RI, et al (2003) DNA-repair genetic polymorphisms and breast cancer risk. Cancer Epidemiol Biomarkers Prev 12: 1200–1204. 14652281

[9] Kumar R, Höglund L, Zhao C, Försti A, Snellman E, et al (2003) Single nucleotide polymorphisms in the XPG gene: determination of role in DNA repair and breast cancer risk. Int J Cancer 103: 671–675. 12494477 10.1002/ijc.10870

[10] Jeon HS, Kim KM, Park SH, Lee SY, Choi JE, et al (2003) Relationship between XPG codon 1104 polymorphism and risk of primary lung cancer. Carcinogenesis 24: 1677–1681. 12869423 10.1093/carcin/bgg120

[11] Sanyal S, Festa F, Sakano S, Zhang Z, Steineck G, et al (2004) Polymorphisms in DNA repair and metabolic genes in bladder cancer. Carcinogenesis 25: 729–734. 14688016 10.1093/carcin/bgh058

[12] Blankenburg S, König IR, Moessner R, Laspe P, Thoms KM, et al (2005) No association between three xeroderma pigmentosum group C and one group G gene polymorphisms and risk of cutaneous melanoma. Eur J Hum Genet 13: 253–255. 15494739 10.1038/sj.ejhg.5201296

[13] Weiss JM, Weiss NS, Ulrich CM, Doherty JA, Voigt LF, et al (2005) Interindividual variation in nucleotide excision repair genes and risk of endometrial cancer. Cancer Epidemiol Biomarkers Prev 14: 2524–2530. 16284373 10.1158/1055-9965.EPI-05-0414

[14] Shen M, Berndt SI, Rothman N, Demarini DM, Mumford JL, et al (2005) Polymorphisms in the DNA nucleotide excision repair genes and lung cancer risk in Xuan Wei, China. Int J Cancer 116: 768–773. 15849729 10.1002/ijc.21117

[15] Bigler J, Ulrich CM, Kawashima T, Whitton J, Potter JD (2005) DNA repair polymorphisms and risk of colorectal adenomatous or hyperplastic polyps. Cancer Epidemiol Biomarkers Prev 14: 2501–2508. 16284370 10.1158/1055-9965.EPI-05-0270

[16] Sakiyama T, Kohno T, Mimaki S, Ohta T, Yanagitani N, et al (2005) Association of amino acid substitution polymorphisms in DNA repair genes TP53, POLI, REV1 and LIG4 with lung cancer risk. Int J Cancer 114: 730–737. 15609317 10.1002/ijc.20790

[17] Cui Y, Morgenstern H, Greenland S, Tashkin DP, Mao J, et al (2006) Polymorphism of Xeroderma Pigmentosum group G and the risk of lung cancer and squamous cell carcinomas of the oropharynx, larynx and esophagus. Int J Cancer 118: 714–720. 16094634 10.1002/ijc.21413

[18] Zienolddiny S, Campa D, Lind H, Ryberg D, Skaug V, et al (2006) Polymorphisms of DNA repair genes and risk of non-small cell lung cancer. Carcinogenesis 27: 560–567. 16195237 10.1093/carcin/bgi232

[19] Millikan RC, Hummer A, Begg C, Player J, de Cotret AR, et al (2006) Polymorphisms in nucleotide excision repair genes and risk of multiple primary melanoma: the Genes Environment and Melanoma Study. Carcinogenesis 27: 610–618. 16258177 10.1093/carcin/bgi252

[20] Mechanic LE, Millikan RC, Player J, de Cotret AR, Winkel S, et al (2006) Polymorphisms in nucleotide excision repair genes, smoking and breast cancer in African Americans and whites: a population-based case–control study. Carcinogenesis 27: 1377–1385. 16399771 10.1093/carcin/bgi330

[21] Huang WY, Berndt SI, Kang D, Chatterjee N, Chanock SJ, et al (2006) Nucleotide excision repair gene polymorphisms and risk of advanced colorectal adenoma: XPC polymorphisms modify smoking-related risk. Cancer Epidemiol Biomarkers Prev 15: 306–311. 16492920 10.1158/1055-9965.EPI-05-0751

[22] García-Closas M, Malats N, Real FX, Welch R, Kogevinas M, et al (2006) Genetic variation in the nucleotide excision repair pathway and bladder cancer risk. Cancer Epidemiol Biomarkers Prev 15: 536–542. 16537713 10.1158/1055-9965.EPI-05-0749

[23] Moreno V, Gemignani F, Landi S, Gioia-Patricola L, Chabrier A, et al (2006) Polymorphisms in genes of nucleotide and base excision repair: risk and prognosis of colorectal cancer. Clin Cancer Res 12: 2101–2108. 16609022 10.1158/1078-0432.CCR-05-1363

[24] Shen M, Zheng T, Lan Q, Zhang Y, Zahm SH, et al (2006) Polymorphisms in DNA repair genes and risk of non-Hodgkin lymphoma among women in Connecticut. Hum Genet 119: 659–668. 16738949 10.1007/s00439-006-0177-2

[25] Shen J, Desai M, Agrawal M, Kennedy DO, Senie RT, et al (2006) Polymorphisms in nucleotide excision repair genes and DNA repair capacity phenotype in sisters discordant for breast cancer. Cancer Epidemiol Biomarkers Prev 15: 1614–1619. 16985021 10.1158/1055-9965.EPI-06-0218

[26] Wen SX, Tang PZ, Zhang XM, Zhao D, Guo YL, et al (2006) Association between genetic polymorphism in xeroderma pigmentosum G gene and risks of laryngeal and hypopharyngeal carcinomas. Zhongguo Yi Xue Ke Xue Yuan Xue Bao 28: 703–706. 17121236

[27] Li C, Hu Z, Liu Z, Wang LE, Strom SS, et al (2006) Polymorphisms in the DNA repair genes XPC, XPD, and XPG and risk of cutaneous melanoma: a case–control analysis. Cancer Epidemiol Biomarkers Prev 15: 2526–2532. 17164380 10.1158/1055-9965.EPI-06-0672

[28] Wu X, Gu J, Grossman HB, Amos CI, Etzel C, et al (2006) Bladder cancer predisposition: a multigenic approach to DNA-repair and cell-cycle-control genes. Am J Hum Genet 78: 464–479. 16465622 10.1086/500848 PMC1380289

[29] Sugimura T, Kumimoto H, Tohnai I, Fukui T, Matsuo K, et al (2006) Gene-environment interaction involved in oral carcinogenesis: molecular epidemiological study for metabolic and DNA repair gene polymorphisms. J Oral Pathol Med 35: 11–18. 16393248 10.1111/j.1600-0714.2005.00364.x

[30] Thirumaran RK, Bermejo JL, Rudnai P, Gurzau E, Koppova K, et al (2006) Single nucleotide polymorphisms in DNA repair genes and basal cell carcinoma of skin. Carcinogenesis 27: 1676–1681. 16501254 10.1093/carcin/bgi381

[31] Hill DA, Wang SS, Cerhan JR, Davis S, Cozen W, et al (2006) Risk of non-Hodgkin lymphoma (NHL) in relation to germline variation in DNA repair and related genes. Blood 108: 3161–3167. 16857995 10.1182/blood-2005-01-026690 PMC1895525

[32] Crew KD, Gammon MD, Terry MB, Zhang FF, Zablotska LB, et al (2007) Polymorphisms in nucleotide excision repair genes, polycyclic aromatic hydrocarbon-DNA adducts, and breast cancer risk. Cancer Epidemiol Biomarkers Prev 16: 2033–2041. 17932351 10.1158/1055-9965.EPI-07-0096

[33] Jorgensen TJ, Visvanathan K, Ruczinski I, Thuita L, Hoffman S, et al (2007) Breast cancer risk is not associated with polymorphic forms of xeroderma pigmentosum genes in a cohort of women from Washington County, Maryland. Breast Cancer Res Treat 101: 65–71. 16823510 10.1007/s10549-006-9263-3

[34] Romanowicz-Makowska H, Smolarz B, Kulig A (2007) Polymorphisms in XRCC1 and ERCC4/XPF DNA repair genes and associations with breast cancer risk in women. Pol Merkur Lekarski 22: 200–203. 17682675

[35] Povey JE, Darakhshan F, Robertson K, Bisset Y, Mekky M, et al (2007) DNA repair gene polymorphisms and genetic predisposition to cutaneous melanoma. Carcinogenesis 28: 1087–1093. 17210993 10.1093/carcin/bgl257

[36] Wang LE, Li C, Strom SS, Goldberg LH, Brewster A, et al (2007) Repair capacity for UV light induced DNA damage associated with risk of nonmelanoma skin cancer and tumor progression. Clin Cancer Res 13: 6532–6539. 17975167 10.1158/1078-0432.CCR-07-0969

[37] Shen M, Purdue MP, Kricker A, Lan Q, Grulich AE, et al (2007) Polymorphisms in DNA repair genes and risk of non-Hodgkin's lymphoma in New South Wales, Australia. Haematologica 92: 1180–1185. 17666372 10.3324/haematol.11324

[38] McWilliams RR, Bamlet WR, Cunningham JM, Goode EL, de Andrade M, et al (2008) Polymorphisms in DNA repair genes, smoking, and pancreatic adenocarcinoma risk. Cancer Res 68: 4928–4935. 18544627 10.1158/0008-5472.CAN-07-5539 PMC2652067

[39] Hooker S, Bonilla C, Akereyeni F, Ahaghotu C, Kittles RA (2008) NAT2 and NER genetic variants and sporadic prostate cancer susceptibility in African Americans. Prostate Cancer Prostatic Dis 11: 349–356. 18026184 10.1038/sj.pcan.4501027

[40] Smith TR, Levine EA, Freimanis RI, Akman SA, Allen GO, et al (2008) Polygenic model of DNA repair genetic polymorphisms in human breast cancer risk. Carcinogenesis 29: 2132–2138. 18701435 10.1093/carcin/bgn193 PMC2722862

[41] Chang JS, Wrensch MR, Hansen HM, Sison JD, Aldrich MC, et al (2008) Nucleotide excision repair genes and risk of lung cancer among San Francisco Bay Area Latinos and African Americans. Int J Cancer 123: 2095–2104. 18709642 10.1002/ijc.23801 PMC2734972

[42] Rajaraman P, Bhatti P, Doody MM, Simon SL, Weinstock RM, et al (2008) Nucleotide excision repair polymorphisms may modify ionizing radiation-related breast cancer risk in US radiologic technologists. Int J Cancer 123: 2713–2716. 18767034 10.1002/ijc.23779 PMC3984912

[43] Frei˘din MB, Ivanina PV, Takhauov RM, Goncharova IA, Dvornichenko MV, et al (2008) The evaluation of association between polymorphisms of DNA excision repair enzyme genes and risk of malignant tumors development in Siberian Group of Chemical Enterprises workers. Radiats Biol Radioecol 48: 439–444. 18825991

[44] Hung RJ, Christiani DC, Risch A, Popanda O, Haugen A, et al (2008) International Lung Cancer Consortium: pooled analysis of sequence variants in DNA repair and cell cycle pathways. Cancer Epidemiol Biomarkers Prev 17: 3081–3089. 18990748 10.1158/1055-9965.EPI-08-0411 PMC2756735

[45] He X, Ye F, Zhang J, Cheng Q, Shen J, et al (2008) Susceptibility of XRCC3, XPD, and XPG genetic variants to cervical carcinoma. Pathobiology 75: 356–363. 19096231 10.1159/000164220

[46] Pardini B, Naccarati A, Novotny J, Smerhovsky Z, Vodickova L, et al (2008) DNA repair genetic polymorphisms and risk of colorectal cancer in the Czech Republic. Mutat Res 638: 146–153. 17991492 10.1016/j.mrfmmm.2007.09.008

[47] Joshi AD, Corral R, Siegmund KD, Haile RW, Le Marchand L, et al (2009) Red meat and poultry intake, polymorphisms in the nucleotide excision repair and mismatch repair pathways and colorectal cancer risk. Carcinogenesis 30: 472–479. 19029193 10.1093/carcin/bgn260 PMC2722151

[48] El-Zein R, Monroy CM, Etzel CJ, Cortes AC, Xing Y, et al (2009) Genetic polymorphisms in DNA repair genes as modulators of Hodgkin disease risk. Cancer 115: 1651–1659. 19280628 10.1002/cncr.24205 PMC2854485

[49] Wen H, Ding Q, Fang ZJ, Xia GW, Fang J (2009) Population study of genetic polymorphisms and superficial bladder cancer risk in Han-Chinese smokers in Shanghai. Int Urol Nephrol 41: 855–864. 19350405 10.1007/s11255-009-9560-y

[50] Narter KF, Ergen A, Agaçhan B, Görmüs U, Timirci O, et al (2009) Bladder cancer and polymorphisms of DNA repair genes (XRCC1, XRCC3, XPD, XPG, APE1, hOGG1). Anticancer Res 29: 1389–1393. 19414392

[51] Abbasi R, Ramroth H, Becher H, Dietz A, Schmezer P, et al (2009) Laryngeal cancer risk associated with smoking and alcohol consumption is modified by genetic polymorphisms in ERCC5, ERCC6 and RAD23B but not by polymorphisms in five other nucleotide excision repair genes. Int J Cancer 125: 1431–1439. 19444904 10.1002/ijc.24442

[52] Hussain SK, Mu LN, Cai L, Chang SC, Park SL, et al (2009) Genetic variation in immune regulation and DNA repair pathways and stomach cancer in China. Cancer Epidemiol Biomarkers Prev 18: 2304–2309. 19661089 10.1158/1055-9965.EPI-09-0233 PMC2725326

[53] McKean-Cowdin R, Barnholtz-Sloan J, Inskip PD, Ruder AM, Butler M, et al (2009) Associations between Polymorphisms in DNA Repair Genes and Glioblastoma. Cancer Epidemiology Biomarkers & Prevention 18: 1118–1126. 10.1158/1055-9965.EPI-08-1078 PMC2667563 19318434

[54] Pan J, Lin J, Izzo JG, Liu Y, Xing J, et al (2009) Genetic susceptibility to esophageal cancer: the role of the nucleotide excision repair pathway. Carcinogenesis 30: 785–792. 19270000 10.1093/carcin/bgp058 PMC2675653

[55] Han J, Haiman C, Niu T, Guo Q, Cox DG, et al (2009) Genetic variation in DNA repair pathway genes and premenopausal breast cancer risk. Breast Cancer Res Treat 115: 613–622. 18551366 10.1007/s10549-008-0089-z PMC2693208

[56] Liu Y, Scheurer ME, El-Zein R, Cao Y, Do KA, et al (2009) Association and Interactions between DNA Repair Gene Polymorphisms and Adult Glioma. Cancer Epidemiology Biomarkers & Prevention 18: 204–214. 10.1158/1055-9965.EPI-08-0632 PMC2917049 19124499

[57] Agalliu I, Kwon EM, Salinas CA, Koopmeiners JS, Ostrander EA, et al (2010) Genetic variation in DNA repair genes and prostate cancer risk: results from a population-based study. Cancer Causes Control 21: 289–300. 19902366 10.1007/s10552-009-9461-5 PMC2811225

[58] Rajaraman P, Hutchinson A, Wichner S, Black PM, Fine HA, et al (2010) DNA repair gene polymorphisms and risk of adult meningioma, glioma, and acoustic neuroma. Neuro Oncol 12: 37–48. 20150366 10.1093/neuonc/nop012 PMC2940551

[59] Ming-Shiean H, Yu JC, Wang HW, Chen ST, Hsiung CN, et al (2010) Synergistic effects of polymorphisms in DNA repair genes and endogenous estrogen exposure on female breast cancer risk. Ann Surg Oncol 17: 760–771. 20183911 10.1245/s10434-009-0802-0

[60] Li LM, Zeng XY, Ji L, Fan XJ, Li YQ, et al (2010) Association of XPC and XPG polymorphisms with the risk of hepatocellular carcinoma. Zhonghua Gan Zang Bing Za Zhi 18: 271–275. 20460046 10.3760/cma.j.issn.1007-3418.2010.04.009

[61] Canbay E, Agachan B, Gulluoglu M, Isbir T, Balik E, et al (2010) Possible associations of APE1 polymorphism with susceptibility and HOGG1 polymorphism with prognosis in gastric cancer. Anticancer Res 30: 1359–1364. 20530453

[62] Figl A, Scherer D, Nagore E, Bermejo JL, Botella-Estrada R, et al (2010) Single-nucleotide polymorphisms in DNA-repair genes and cutaneous melanoma. Mutation Research - Genetic Toxicology and Environmental Mutagenesis 702: 8–16. 20601096 10.1016/j.mrgentox.2010.06.011

[63] Rouissi K, Bahria IB, Bougatef K, Marrakchi R, Stambouli N, et al (2011) The effect of tobacco, XPC, ERCC2 and ERCC5 genetic variants in bladder cancer development. BMC Cancer 11: 101. 21426550 10.1186/1471-2407-11-101 PMC3068124

[64] Liu D, Wu HZ, Zhang YN, Kang H, Sun MJ, et al (2011) DNA repair genes XPC, XPG polymorphisms: Relation to the risk of colorectal carcinoma and therapeutic outcome with oxaliplatin-based adjuvant chemotherapy. Mol Carcinog doi:10.1002/mc.21862 10.1002/mc.21862 22213216

[65] Canbay E, Cakmakoglu B, Zeybek U, Sozen S, Cacina C, et al (2011) Association of APE1 and hOGG1 polymorphisms with colorectal cancer risk in a Turkish population. Curr Med Res Opin 27: 1295–1302. 21561390 10.1185/03007995.2011.573544

[66] Gonçalves FT, Francisco G, de Souza SP, Luiz OC, Festa-Neto C, et al (2011) European ancestry and polymorphisms in DNA repair genes modify the risk of melanoma: a case–control study in a high UV index region in Brazil. J Dermatol Sci 64: 59–66. 21733660 10.1016/j.jdermsci.2011.06.003

[67] Ibarrola-Villava M, Peña-Chilet M, Fernandez LP, Aviles JA, Mayor M, et al (2011) Genetic polymorphisms in DNA repair and oxidative stress pathways associated with malignant melanoma susceptibility. European Journal of Cancer 47: 2618–2625. 21641795 10.1016/j.ejca.2011.05.011

[68] Doherty JA, Weiss NS, Fish S, Fan W, Loomis MM, et al (2011) Polymorphisms in nucleotide excision repair genes and endometrial cancer risk. Cancer Epidemiol Biomarkers Prev 20: 1873–1882. 21750170 10.1158/1055-9965.EPI-11-0119 PMC3169742

[69] Biason P, Hattinger CM, Innocenti F, Talamini R, Alberghini M, et al (2012) Nucleotide excision repair gene variants and association with survival in osteosarcoma patients treated with neoadjuvant chemotherapy. Pharmacogenomics J 12: 476–483. 21826087 10.1038/tpj.2011.33 PMC3935514

[70] Krupa R, Kasznicki J, Gajęcka M, Rydzanicz M, Kiwerska K, et al (2011) Polymorphisms of the DNA repair genes XRCC1 and ERCC4 are not associated with smoking- and drinking-dependent larynx cancer in a Polish population. Exp Oncol 33: 55–56. 21423097

[71] Yu H, Liu Z, Huang YJ, Yin M, Wang LE, et al (2012) Association between single nucleotide polymorphisms in ERCC4 and risk of squamous cell carcinoma of the head and neck submitted. PLoS One 7: e41853. 22848636 10.1371/journal.pone.0041853 PMC3407112

[72] Ma H, Yu H, Liu Z, Wang LE, Sturgis EM, et al (2012) Polymorphisms of XPG/ERCC5 and risk of squamous cell carcinoma of the head and neck. Pharmacogenet Genomics 22: 50–57. 22108238 10.1097/FPC.0b013e32834e3cf6 PMC3237901

[73] Gil J, Ramsey D, Stembalska A, Karpinski P, Pesz KA, et al (2012) The C/A polymorphism in intron 11 of the XPC gene plays a crucial role in the modulation of an individual's susceptibility to sporadic colorectal cancer. Mol Biol Rep 39: 527–534. 21559836 10.1007/s11033-011-0767-5

[74] Berhane N, Sobti RC, Mahdi SA (2012) DNA repair genes polymorphism (XPG and XRCC1) and association of prostate cancer in a north Indian population. Mol Biol Rep 39: 2471–2479. 21670956 10.1007/s11033-011-0998-5

[75] Paszkowska-Szczur K, Scott RJ, Serrano-Fernandez P, Mirecka A, Gapska P, et al (2013) Xeroderma pigmentosum genes and melanoma risk. Int J Cancer 133: 1094–1100. 23436679 10.1002/ijc.28123

[76] Weiss JM, Weiss NS, Ulrich CM, Doherty JA, Chen C (2006) Nucleotide excision repair genotype and the incidence of endometrial cancer: effect of other risk factors on the association. Gynecol Oncol 103: 891–896. 16806437 10.1016/j.ygyno.2006.05.020

[77] Weiss JM, Weiss NS, Ulrich CM, Doherty JA, Voigt LF, et al (2005) Interindividual variation in nucleotide excision repair genes and risk of endometrial cancer. Cancer Epidemiol Biomarkers Prev 14: 2524–2530. 16284373 10.1158/1055-9965.EPI-05-0414

[78] Smith TR, Liu-Mares W, Van Emburgh BO, Levine EA, Allen GO, et al (2011) Genetic polymorphisms of multiple DNA repair pathways impact age at diagnosis and TP53 mutations in breast cancer. Carcinogenesis 32: 1354–1360. 21700777 10.1093/carcin/bgr117 PMC3165125

[79] Zienolddiny S, Campa D, Lind H, Ryberg D, Skaug V, et al (2006) Polymorphisms of DNA repair genes and risk of non-small cell lung cancer. Carcinogenesis 27: 560–567. 16195237 10.1093/carcin/bgi232

[80] Wen H, Feng CC, Fang ZJ, Xia GW, Jiang HW, et al (2013) Study on Bladder Cancer Susceptibility and Genetic Polymorphisms of XPC, XPG, and CYP in Smokers and Non-Smokers. Actas Urol Esp 37: 259–265. 23246108 10.1016/j.acuro.2012.04.007

[81] Wang XF, Liu S, Shao ZK (2013) Effects of polymorphisms in nucleotide excision repair genes on glioma risk in a Chinese population. Gene doi:pii: S0378-1119(13)00896-2. 10.1016/j.gene.2013.07.025 10.1016/j.gene.2013.07.025 23911298

[82] Santos LS, Gomes BC, Gouveia R, Silva SN, Azevedo AP, et al (2013) The role of CCNH Val270Ala (rs2230641) and other nucleotide excision repair polymorphisms in individual susceptibility to well-differentiated thyroid cancer. Oncol Rep 30: 2458–2466. 23982724 10.3892/or.2013.2702

[83] Cheng HB, Xie C, Zhang RY, Hu SS, Wang Z, et al (2013) Xeroderma pigmentosum complementation group of polymorphisms influence risk of glioma. Asian Pac J Cancer Prev 14: 4083–4087. 23991957 10.7314/apjcp.2013.14.7.4083

[84] Zhu ML, Wang M, Cao ZG, He J, Shi TY, et al (2012) Association between the ERCC5 Asp1104His polymorphism and cancer risk: a meta-analysis. PLoS One 7: e36293. 22815677 10.1371/journal.pone.0036293 PMC3399856

[85] Shi TY, He J, Qiu LX, Zhu ML, Wang MY, et al (2012) Association between XPF polymorphisms and cancer risk: a meta-analysis. PLoS One 7: e38606. 22768293 10.1371/journal.pone.0038606 PMC3388076

[86] Davey SG, Egger M (1997) Meta-analyses of randomized controlled trials. Lancet 350: 1182. 9343537 10.1016/s0140-6736(05)63833-0

[87] Higgins JP, Thompson SG, Deeks JJ, Altman DG (2003) Measuring inconsistency in meta-analysis. Br Med J 327: 557–560. 12958120 10.1136/bmj.327.7414.557 PMC192859

[88] Mantel N, Haenszel W (1959) Statistical aspects of the analysis of data from retrospective studies of disease. Natl Cancer Inst 22: 719–748. 13655060

[89] DerSimonian R, Laird N (1986) Meta-analysis in clinical trials. Control Clin Trials 7: 177–188. 3802833 10.1016/0197-2456(86)90046-2

[90] Klug SJ, Ressing M, Koenig J, Abba MC, Agorastos T, et al (2009) TP53 codon 72 polymorphism and cervical cancer: a pooled analysis of individual data from 49 studies. Lancet Oncol 10: 772–784. 19625214 10.1016/S1470-2045(09)70187-1

[91] Begg CB, Mazumdar M (1994) Operating characteristics of a rank correlation test for publication bias. Biometrics 50: 1088–1101. 7786990

[92] Egger M, Smith DG, Schneider M, Minder C (1997) Bias in meta-analysis detected by a simple, graphical test. Br Med J 315: 629–634. 9310563 10.1136/bmj.315.7109.629 PMC2127453

[93] Dual S, Tweedie R (2000) A nonparametric “trim and fill” method of accounting for publication bias in meta-analysis. J Am Stat Assoc 95: 89–98.

[94] Higgins JPT, Green S (2008) Cochrane handbook for systematic reviews of interventions version 5.0.1. The Cochrane Collaboration, Oxford.

[95] Visapää JP, Götte K, Benesova M, Li J, Homann N, et al (2004) Increased cancer risk in heavy drinkers with the alcohol dehydrogenase 1C*1 allele, possibly due to salivary acetaldehyde. Gut 53: 871–876. 15138216 10.1136/gut.2003.018994 PMC1774061

